# Essential updates 2020/2021: Advancing precision medicine for comprehensive rectal cancer treatment

**DOI:** 10.1002/ags3.12646

**Published:** 2022-12-27

**Authors:** Ichiro Takemasa, Atsushi Hamabe, Masaaki Miyo, Emi Akizuki, Koichi Okuya

**Affiliations:** ^1^ Department of Surgery, Surgical Oncology and Science Sapporo Medical University Sapporo Japan; ^2^ Department of Gastroenterological Surgery, Graduate School of Medicine Osaka University Osaka Japan

**Keywords:** diagnosis, multimodal therapy, rectal cancer, stratification, surgery

## Abstract

In the paradigm shift related to rectal cancer treatment, we have to understand a variety of new emerging topics to provide appropriate treatment for individual patients as precision medicine. However, information on surgery, genomic medicine, and pharmacotherapy is highly specialized and subdivided, creating a barrier to achieving thorough knowledge. In this review, we summarize the perspective for rectal cancer treatment and management from the current standard‐of‐care to the latest findings to help optimize treatment strategy.

## Graphical abstract

Regarding rectal cancer treatment, information on surgery, genomic medicine, and pharmacotherapy is highly specialized and subdivided, creating a barrier to achieving thorough knowledge. In this review, we summarize the perspective for rectal cancer treatment and management from the current standard‐of‐care to the latest findings to help optimize treatment strategy.

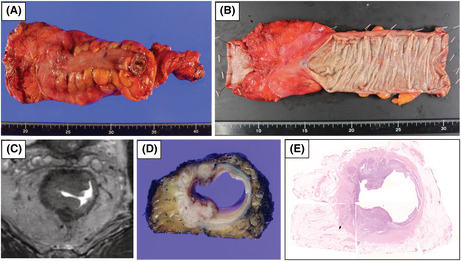

## 1 INTRODUCTION

An estimated 1.9 million cases of colorectal cancer occurred in 2020, with rectal cancer accounting for roughly 40% of cases.^1^ The incidence is especially high in developed countries.^1^ Rectal cancer is difficult to cure, likely due to the technical difficulty of the surgery or the high recurrence rate compared to colon cancer. This difference is reflected in the separate guidelines specific for rectal cancer in Western countries.^2^, ^3^

Particularly for the treatment of locally advanced rectal cancer (LARC), much progress has been made for more than two decades, including diagnostic technology, advancements in surgery, and developments in multimodal therapy. In the past, postoperative local recurrence had been the greatest challenge in LARC. Now, however, the rate of local recurrence has declined dramatically, mainly due to the prevalence of total mesorectal excision (TME) and the adoption of multimodal therapy. The current topic of discussion is how we can control distant metastasis. A novel treatment approach, total neoadjuvant therapy (TNT), has emerged to handle this issue, with promising results in some recent reports. From the technical aspect of surgery, novel technologies have enabled us to see things that had been invisible, such as the near‐infrared region (NIR) spectrometry, or liquid biopsy. We may be facing a new era of rectal cancer treatment. In this review, we discuss the evidence to develop an understanding of the whole picture of rectal cancer treatment, thereby indicating future trends.

## 2 SURGERY FOR RECTAL CANCER

### 2.1 Evolution of minimally invasive surgery

Modern surgery for colorectal cancer has continuously evolved since the introduction of abdominoperineal resection by Miles, along with the establishment of anesthesia and infection control. Laparoscopic surgery for colon cancer is now widely used to reduce the surgical invasiveness compared to open surgery, mainly based on major randomized clinical trials (RCTs), including the COLOR, CLASICC, and JCOG0404 trials.^4^, ^5^, ^6^, ^7^, ^8^ Although the efficacy of laparoscopic surgery for rectal cancer in phase II trials has led to its widespread adoption, the efficacy of laparoscopic surgery for LARC remains controversial.^9^, ^10^, ^11^ Two large RCTs, the COLOR II and COREAN trials, have shown similar short‐term pathological and 3‐y survival outcomes between open surgery and laparoscopic surgery for LARC, whereas two other RCTs, ACOSOG Z6051 and ALaCaRT, failed to demonstrate noninferiority of laparoscopic surgery to open surgery with regard to composite pathological endpoints, including the quality of TME and circumferential resection margin (CRM). These results suggest that there may be a concern that manipulation in the deep pelvis with straight‐shaped laparoscopic forceps surgery is too difficult to safely and securely dissect the correct plane around the tumor with sufficient margin.^12^, ^13^, ^14^, ^15^ On the other hand, increasing demand for patient satisfaction with surgery, including better cosmetic and functional outcomes, have raised the social need for minimally invasive surgery.^16^, ^17^, ^18^, ^19^ Robotic systems are a promising advanced technology that could overcome some of the inherent limitations of laparoscopic surgery for rectal cancer, providing high‐quality three‐dimensional images, articulating instruments, stable camera work, and motion scale function.^20^, ^21^ The ROLARR trial was performed in a multicenter setting to compare robotic‐assisted and conventional laparoscopic rectal cancer surgery, indicating that robotic‐assisted laparoscopic surgery did not significantly reduce the risk of conversion to open laparotomy (8.1% vs 12.2%).^22^ However, a retrospective cohort study based on the National Clinical Database in Japan found a significantly lower conversion rate to open surgery in robot‐assisted laparoscopic surgery than in conventional laparoscopic low anterior resection (0.7% vs 2.0%), less intraoperative blood loss (15 ml vs 20 ml), and a shorter postoperative hospital stay (13 d vs 14 d).^23^ Regarding the long‐term results of robot‐assisted laparoscopic surgery, the oncological differences between robot‐assisted and conventional laparoscopic rectal cancer surgery have not yet been reported in RCTs, although the REAL trial, an RCT comparing the two procedures, recently demonstrated that CRM, which is a surrogate marker of oncological outcome, was more favorable with the robotic approach.^24^ We are also currently carrying out the VITRUVIANO trial, in which the results of CRM using the robotic approach in Japanese patients are being analyzed. According to the recent meta‐analysis, the prognosis, including overall survival and local or distant recurrence rate, is comparable.^25^, ^26^ Although the outcomes of robot‐assisted laparoscopic surgery remain controversial, a recent meta‐analysis indicated that robot‐assisted surgery with advanced visualization can improve autonomic nerve preservation, thereby providing better urinary and erectile function than conventional laparoscopic surgery for rectal cancer patients.^27^ We consider that robotic surgery can meet patient demands for function preservation, especially in the era of minimally invasive surgery. However, the usefulness of robotic surgery has to be validated from multiple perspectives.

### 2.2 Key surgical concepts for rectal cancer

Surgical treatment is the mainstay in multidisciplinary treatment for locally advanced rectal cancer, and the quality of surgery is directly associated with postoperative local recurrence. Thus, appropriate objective indicators are needed to assess the quality of surgery. In the 1980s, Heald et al proposed the importance of TME and reported that complete TME leads to a lower local recurrence rate and prolonged overall survival.^28^ Adam et al also reported a significantly increased local recurrence rate in patients with a CRM ≤1 mm.^29^ Since then, TME has become the standard procedure for rectal cancer, and its completion and assurance of CRM are considered the most important surgical factors and used as indicators of surgical quality. In general, CRM is judged as positive if the margin is ≤1 mm. Notably, if the tumor is in close proximity to or involves the mesorectal fascia, the CRM may be positive even if TME is completed, and accurate preoperative diagnosis is necessary to ensure a negative CRM.

**FIGURE 1:**
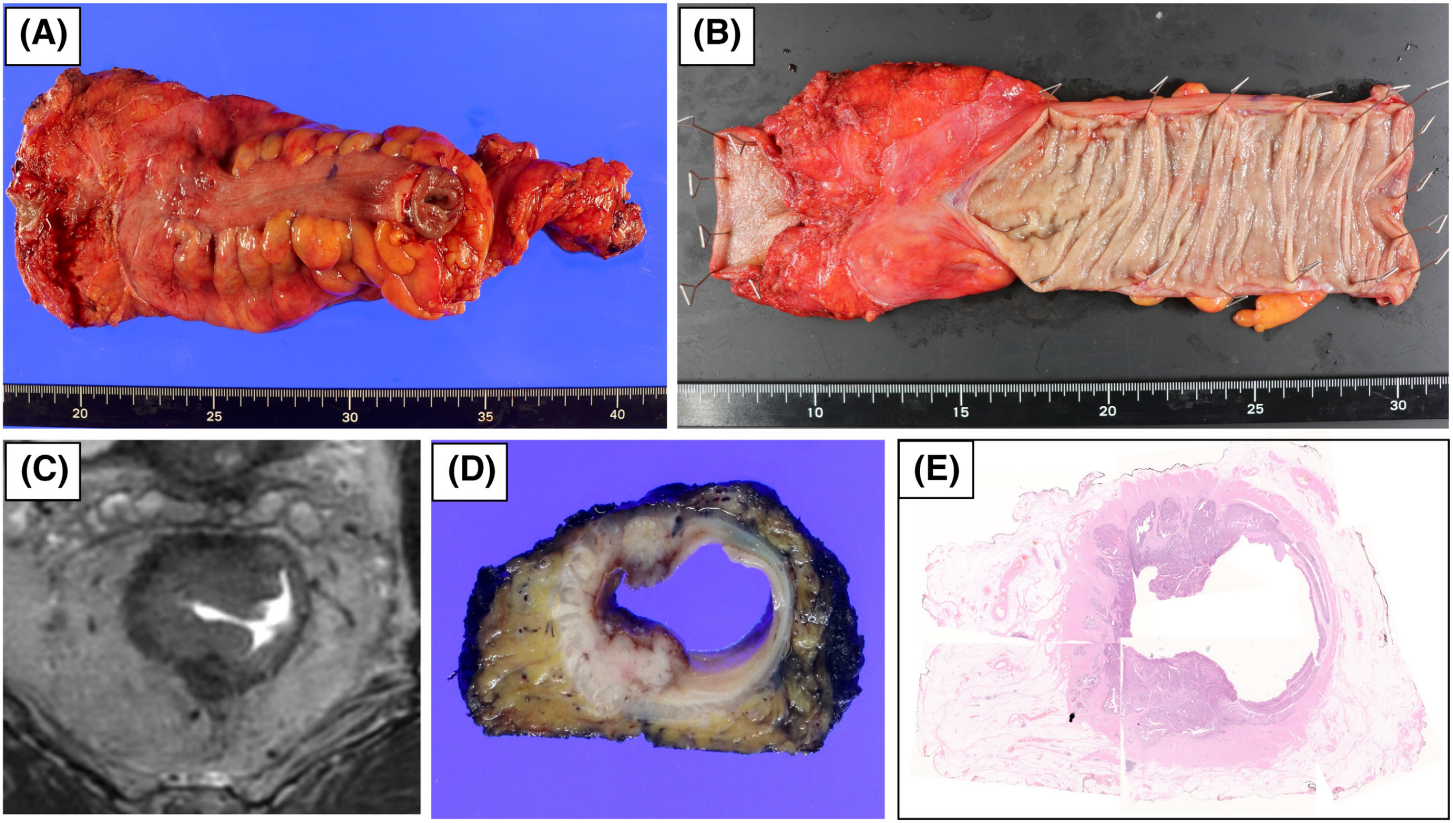
Semiopened circular specimen processing method for pathological circumferential resection margin (CRM) assessment (a) anterior view of the resected rectal specimen. (b) Semiopened rectal specimen. (c) Axial section of rectal cancer on MRI. (d) Transverse section of the semiopened rectal specimen. (e) Hematoxylin and eosin staining of the transverse section, allowing a comparison of the pathological CRM and preoperative mesorectal fascia (MRF) involvement

In Western countries, CRM and TME, especially CRM are used as the most important indicators of the oncological quality of surgery but, in Japan, these indicators have not been regarded as important for a long time. According to Japanese practice, the resected specimen is opened longitudinally to assess the tumor morphology and distal resection margin, and the mesentery is removed from the specimen to ascertain the number and location of lymph node metastases (LNMs), making accurate assessment of the CRM difficult. Therefore, it has long been difficult to compare the quality of surgery in Japan with the quality in Western countries using CRM. For the purpose of validating the results of Japanese MIS, the development of a method to assess CRM and TME in Japan has been desired. We previously developed the “semi‐opened circular specimen processing method” for pathological CRM assessment that fits into Japanese practice (Figure 1),^30^ which was verified by a multicenter validity study.^31^ Using this method, the oncological validity of laparoscopic surgery for advanced rectal cancer in Japan was assessed in the PRODUCT study, which demonstrated that the positive CRM rate was 8.6%.^32^ Table 1 compares the positive CRM rates between four representative RCTs and the PRODUCT study. Regarding robotic surgery, the VITRUVIANO trial, a prospective, multicenter, registry study of the oncological validity of robot‐assisted surgery for advanced rectal cancer, is currently underway, in which the primary endpoint is CRM by the semiopened circular specimen processing method. For quality control, the operating surgeons are certified if the experience with robotic surgeries is more than at least 40 cases. Enrollment is already completed and the positive CRM rate after robotic surgery in Japan is going to be clarified in the near future.

**TABLE 1:** Comparison of the results of laparoscopic surgery in randomized controlled trials and the PRODUCT study analyzing the rate of CRM positivity

Trial			COLOR II^12^	COREAN^13^	ALaCaRT^15^	Z6051^14^	PRODUCT^32^
Definition of positive CRM			<2 mm	≤1 mm	<1 mm	≤1 mm	≤1 mm
Eligibility							
	Stage or T factor		T1‐T3	T3N0 or any T N1‐2	T1‐T3 with clear CRM	Stage II/III	Stage II/III
	Distance from AV, cm		15	9	15	12	12
Median age, y			67	58	65	58	66
Male sex			448 (64%)	110 (65%)	160 (67%)	156 (65%)	202 (67%)
Median BMI, kg/m^2^			26	24	27	26	22
Preoperative therapy		CRT	412 (59%)	100%	119 (50%)	235 (98%)	27 (9%)
		NAC	196 (32%)	0	0	4 (1.7%)	45 (15%)
		TNT	N/A	N/A	N/A	N/A	18 (6%)
Pathological stage		0	33 (5%)	N/A	N/A	55 (23%)	14 (5%)
		I	231 (34%)	N/A	N/A	76 (32%)	2 (<1%)
		II	180 (26%)	N/A	N/A	47 (20%)	76 (25%)
		III	233 (34%)	N/A	N/A	60 (25%)	104 (33%)
		IV	4 (<1%)	N/A	N/A	1 (<1%)	107 (35%)
Pathological T stage		pT0	N/A	40 (24%)	33 (14%)	N/A	16 (5%)
		pTis	N/A	5 (3%)	―	N/A	2 (<1%)
		pT1	N/A	9 (5%)	23 (10%)	N/A	10 (3%)
		pT2	N/A	41 (24%)	67 (28%)	N/A	84 (28%)
		pT3	N/A	73 (43%)	104 (44%)	N/A	176 (59%)
		pT4	N/A	2 (1%)	11 (5%)	N/A	15 (5%)
Pathological N stage		pN0	N/A	135 (79%)	148 (62%)	N/A	196 (65%)
		pN1	N/A	18 (11%)	67 (28%)	N/A	74 (24%)
		pN2	N/A	17 (10.0%)	23 (10%)	N/A	33 (11%)
Rate for CRM positivity			56 (9.5%)	5 (2.9%)	16 (6.7%)	29 (12.1%)	26 (8.6%)

*Note*: Values are given as *n* (%) unless otherwise noted.

### 2.3 Transanal minimally invasive surgery

During laparoscopic surgery for rectal cancer, the transabdominal manipulation in the deep pelvis is technically demanding due, in part, to limited maneuverability in the confined space far from the abdominal wall or the confliction between the forceps. Given that the noninferiority of laparoscopic surgery to open surgery was not demonstrated in the ALaCaRT or ACOSOG Z6051 trials, there may be a concern that this difficulty can deteriorate the quality of TME or jeopardize the acquisition of a clear CRM.

**FIGURE 2:**
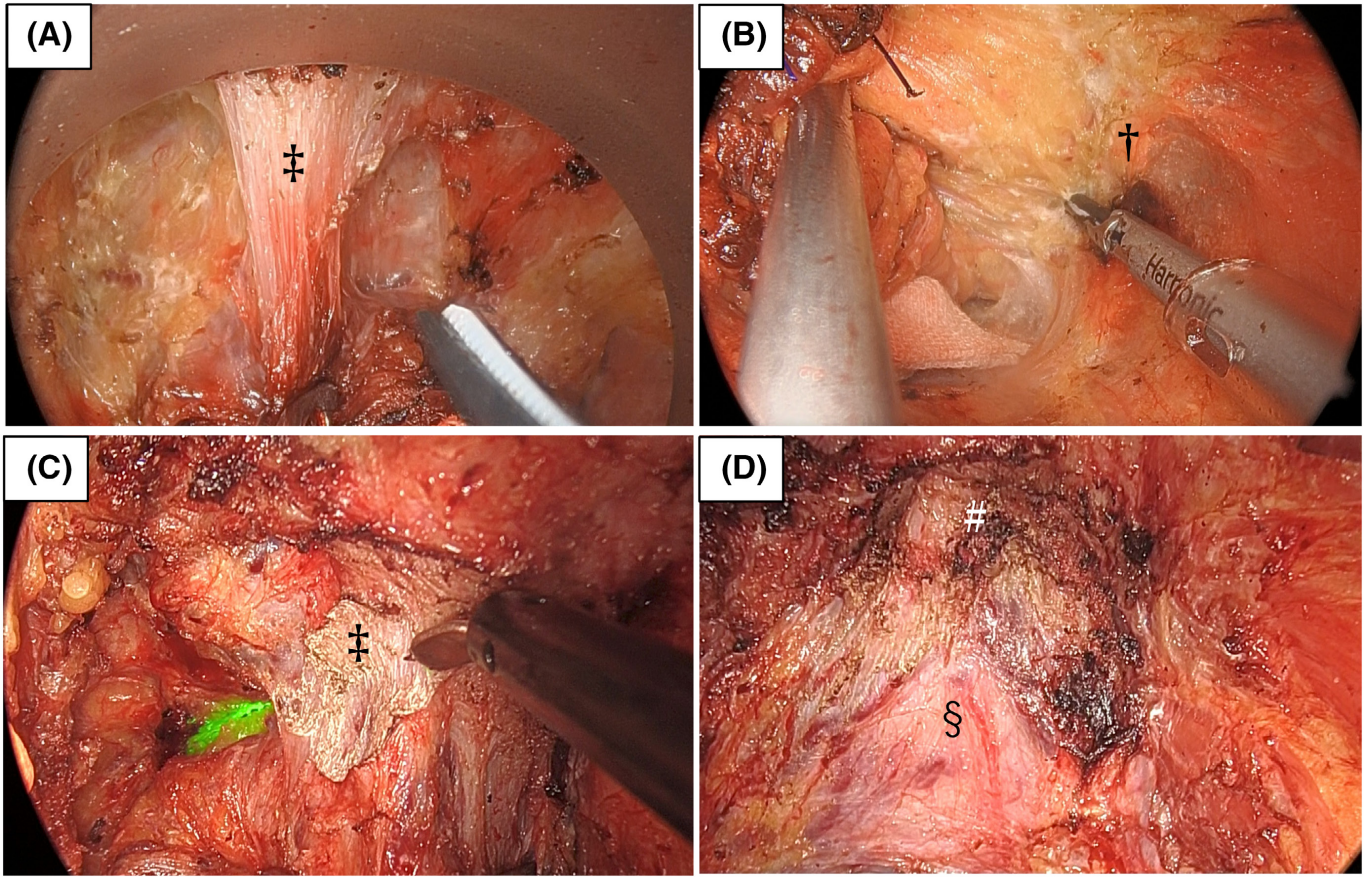
Intraoperative images during transanal total mesorectal excision (TME) and transperineal abdominoperineal excision (APE) (a) dissection of the rectourethral muscle and (b) exposure of pelvic splanchnic nerve S4 in transanal TME. (c) Dissection of the rectourethral muscle and (d) surgical view seen from the perineal side after rectal resection in transperineal APE. ‡rectourethral muscle, †pelvic splanchnic nerve S4, §prostate, #urethra

In 2010, Sylla et al reported the application of transanal TME (TaTME) for rectal cancer resection to address the above‐mentioned difficulties.^33^, ^34^ During TaTME, the operating surgeon can perform dissection near the access device placed at the anal canal and the forceps can easily be applied in the direction of the rectal axis, providing better accessibility to the deep pelvis (Figure 2). The following evidence from advanced institutions in this field demonstrates the favorable results of TaTME, with a lower positive CRM rate, improved quality of the TME, or curtailed operative time compared to the conventional laparoscopic approach.^35^, ^36^, ^37^

Compared to conventional surgery, TaTME has shown better oncological results with a definitive distal margin and lower positive CRM rate.^38^ Furthermore, TaTME has been reported to improve safety and functional preservation, with lower conversion rates, fewer postoperative complications, and higher rates of anal preservation.^39^, ^40^, ^41^ In abdominoperineal excision (APE), perineal dissection is conventionally carried out under direct view. However, the anatomical configurations of the pelvis are highly complicated, especially around the rectal anterior wall; thus, the surgeons are forced to deal with this complex area through a restricted wound.^42^ Transperineal APE (TpAPE) has emerged as an advanced approach to TaTME, and it can address the technical difficulty encountered during a perineal procedure (Figure 2).^43^, ^44^

In contrast to the advantages of TaTME, several concerns specific to TaTME should be noted. The first concern is urethral injury, which rarely occurs during the transabdominal approach. Reportedly, urethral injury occurs in ~1% of procedures and is especially frequent during the first eight cases of implementation.^45^, ^46^ To avoid urethral injury, understanding of the pelvic anatomy from the perineal side is mandatory for the surgeons to dissect the rectal anterior wall correctly. The second concern is that TaTME has a possibility of increasing the local recurrence rate and promoting a multifocal pattern of recurrence. A Norwegian national audit estimated a rate of local recurrence at 2.4 y of 11.6%, with a multifocal or extensive pattern of local recurrence in two‐thirds of patients.^47^ Similarly, according to the Dutch study, multifocal recurrence was frequent during the implementation phase of TaTME.^48^ Presumably, the unfavorable results were caused by leakage of gas or liquid containing malignant cells due to incomplete closure of the purse‐string suture or rectal perforation. In advanced centers, the rate of local recurrence was low (2.0%–3.4%) and a multifocal pattern of recurrence was not found.^39^, ^49^, ^50^ Thus, quality control of TaTME is crucial to securely perform rectal cancer surgery, and the training curriculum should be carried out to introduce TaTME.^51^, ^52^ Ongoing RCTs comparing laparoscopic TME and TaTME, such as COLOR III (NCT02736942) and GRECCAR 11 (NCT02584985), will clarify the validity of TaTME.^53^, ^54^ Furthermore, it is important to address the efficacy of TaTME for far advanced cases, such as combined resection of adjacent organs or total pelvic exenteration.

### 2.4 Lateral lymph node dissection

In lower rectal cancer with a depth of cT3 or cT4, 15%–20% of patients have metastasis in the lateral lymph nodes.^55^ The Japanese standard of care includes lateral lymph node dissection (LLND) in addition to TME, whereas preoperative chemoradiotherapy (CRT) is commonly used in Western countries, and the local recurrence rates for both are comparable.^56^ JCOG0212 evaluated the noninferiority of mesorectal excision (ME) alone to ME + LLND in stages II and III lower rectal cancer patients without lateral LNM on preoperative imaging. The primary endpoint of 5‐y recurrence‐free survival was not proven to be noninferior to ME + LLND, and the local recurrence rate was significantly reduced from 12.6% to 7.4% after LLND,^57^ indicating that LLND could be effective in preventing local recurrence. However, LLND is associated with longer operative times, increased blood loss, and risk of functional impairment. Furthermore, relapse‐free survival did not differ whether LLND was or was not performed; therefore, more than a few Japanese surgeons even omit LLND for prophylactic purposes and restrict the indication of LLND to cases with evident metastasis. In contrast, in cases with enlarged lateral lymph nodes, even if neoadjuvant CRT is administered, the addition of LLND could prevent lateral local recurrence, suggesting that LLND is an important option in multimodal therapy and would be effective in select cases.^58^, ^59^ Autonomic nerve‐preserving LLND, which was developed in Japan, is now being appreciated in the West, and the West meets East concept is attracting a lot of attention.^60^

Given that intensified multimodal treatment combined with LLND can be effective at lowering the rate of local recurrence for a portion of patients, more accurate criteria are needed for predicting metastasis to lateral lymph nodes. Several studies have demonstrated the risk factors for metastasis, including the size or shape of the lateral lymph node, tumor location, or extramural venous invasion (EMVI).^61^, ^62^ EMVI is defined as the active invasion of malignant cells into veins beyond the muscularis propria in colorectal cancer that can be diagnosed on magnetic resonance imaging (MRI).^63^ The combination of EMVI with the size of the lateral lymph node could differentiate high‐risk cases, which may optimize the indication for LLND.^61^, ^62^

Concerning LLND techniques, the conventional procedure has been open surgery, although the indication for MIS has been gradually expanded to LLND. Several retrospective studies have demonstrated the safety and feasibility of laparoscopic LLND.^64^, ^65^ To enhance the precision of the operation in the deep and narrow cavity, application of robotic surgery for LLND is being attempted, demonstrating that it may decrease postoperative complications compared to open or laparoscopic surgery^66^, ^67^ or significantly improve the 5‐y local recurrence‐free survival rate compared to open surgery.^68^ The deepest lymph node station, 263, is reportedly the most frequently metastatic region, which is technically difficult to dissect.^69^ We consider that robotic LLND can be efficacious to clear the lateral lymph nodes, including station 263.

## 3 MEASUREMENTS TO PREVENT RECTAL CANCER SURGERY‐RELATED COMPLICATIONS

A variety of complications can occur after rectal cancer resection, including anastomotic leakage (AL), infection, bowel obstruction or ileus, and surgical site infection. In this chapter we discuss how we efficiently prevent AL or AL‐related problems, considering its significance in clinical practice.

### 3.1 ICG fluorescence angiography

**FIGURE 3:**
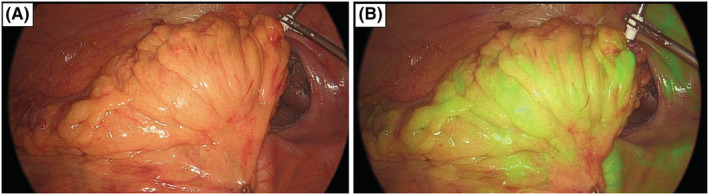
Evaluation of vascular perfusion using indocyanine green (ICG) fluorescence angiography. View with near‐infrared (NIR) light before (a) and after (b) ICG injection. Vascular perfusion was considered good when it was well visualized within 60 s

Anastomotic leakage is one of the most detrimental complications of colorectal surgery, which is associated with elevated morbidity and mortality rates, as well as the risk of local recurrence in rectal cancer resection.^70^, ^71^, ^72^, ^73^ Various factors, including surgical, patient, and tumor factors, are associated with the occurrence of AL.^74^, ^75^, ^76^ Adequate blood flow to the anastomotic intestinal stump is also essential to avoid AL, but it is not always easy to precisely evaluate blood flow. Conventionally, intestinal blood flow is evaluated by surgeons using several clinical signs, such as the color of the intestinal mucosa, peristaltic movement, bleeding from the marginal artery, and palpable arterial pulses in the mesentery. These signs are useful for judging the intestinal blood flow, but it may easily be surgeon‐dependent and inconsistent.^77^ Indocyanine green (ICG) fluorescence angiography allows surgeons to visualize intestinal perfusion in real time^78^, ^79^, ^80^ and is anticipated to decrease the incidence of AL (Figure 3).^81^, ^82^, ^83^, ^84^ A meta‐analysis to assess the efficacy and safety of ICG in colorectal cancer surgery in 11 047 patients showed that ICG fluorescence angiography significantly reduces the rate of AL (3.7% with ICG vs 7.6% without ICG), but this meta‐analysis was mostly comprised of retrospective studies and small studies.^85^ In the interim analysis of the PILLAR‐III trial, a randomized, controlled, parallel, multicenter study assessing perfusion outcomes in low anterior resection, the incidence of AL was 9.0% in the ICG group and 9.6% in the non‐ICG group. This trial was terminated because the efficacy was not confirmed.^86^ PILLAR‐III was associated with several limitations: the criteria to assess intestinal blood flow was not standardized, whether the transection line was changed after administering ICG was not clear, and a detailed description of the sample size was lacking. Therefore, the results of PILLAR‐III could not clearly conclude that ICG fluorescence angiography prevented the occurrence of AL.^86^ Currently, RCTs evaluating the effect of ICG angiography on anastomotic leakage in laparoscopic surgery are in progress worldwide, including the EssentiAL study (UMIN:000030240), INTACT (ISCRN:13334746), POSTER (NCT:04012645), AVOID (NCT:04712032), FLUOCOL‐1 (NCT:05168839), and COLORAL (NCT:03602677). The Japanese EssentiAL study has already completed patient enrollment, which was designed after resolving the above‐mentioned limitations of PILLAR‐III. It is anticipated that the results of ongoing RCTs would clearly demonstrate the efficacy of ICG angiography.

### 3.2 Double‐stapling techniques with reinforced linear stapler

Improvements in surgical techniques and development of surgical devices have enabled the double‐stapling technique (DST) and increased sphincter‐preserving surgery. As mentioned in the previous section, reducing AL is crucial to improving rectal cancer outcomes. Tissue damage is one of the risk factors for AL after rectal cancer resection,^87^ and various efforts have been made to reduce tissue damage. Regarding rectal transection, it may be effective to use cartridges appropriate for the transection line and rectal thickness individually, and using a motorized linear stapler may be helpful for avoiding excessive pressure.

Endoscopic evaluation of patients with AL after DST anastomosis has shown that AL often occurs at the intersection site of the circular and linear stapler.^88^ Reinforcement of the intersection may offer a chance to avoid AL, and various methods, including suture reinforcement and bioabsorbable staple line reinforcement, have been validated thus far, although their effectiveness remains unclear.^89^, ^90^ A different method has been developed: the linear stapler with absorbable tissue reinforcement, the application of which has already been reported to be effective for gastrectomy or pancreatectomy.^91^ Based on this background, its use in rectal transection can be considered efficacious in strengthening the intersection. We are currently conducting a multicenter, prospective, observational study (UMIN000030240) to evaluate the safety and efficacy of the reinforced linear stapler in rectal cancer resection. As AL is a multifactorial event, it will require various approaches, among which the use of novel devices for tissue reinforcement may play an important role.

### 3.3 Closure of diverting stoma

Diverting stoma in rectal cancer resection may reduce symptomatic AL or the need for urgent abdominal reoperation for leakage.^92^, ^93^, ^94^ As loop ileostomy is associated with fewer stoma‐related complications than colostomy, loop ileostomy is more commonly constructed in Japan. According to the established guidelines, stomas should be placed away from skin folds, scars, bony prominences, and the belt line.^95^ Moreover, they should be placed within the rectus abdominis muscle and should be visible to the patient. A long distance from the ileocecal valve to the ileostomy is associated with a low risk of stoma retraction and a high risk of ileus, and the height of the distal limb of the ileostomy significantly affects the incidence of parastomal dermatitis and mucocutaneous separation.^96^

Surgical site infection (SSI) is the most common postoperative complication after ileostomy closure, with a reported rate of up to 40%, leading to longer hospital stays and higher medical costs.^97^ Various measures have been taken to reduce the incidence of SSI, such as bowel preparation, the administration of appropriate perioperative antibiotics, wound protection, the use of absorbable sutures, wound irrigation with saline,^98^ and purse‐string skin closure.^97^ We have reported a lower incidence of SSI with primary wound closure and preventive negative‐pressure wound therapy, which may be effective as a preventive option.^99^

## 4 ADVANCED VISUALIZATION

### 4.1 Fluorescence lymph navigation surgery for colorectal cancer

The intraoperative navigation surgery using near‐infrared (NIR) fluorescence has become possible with improvements in laparoscopic surgical devices. In recent years, ICG fluorescence imaging has been applied clinically for real‐time visualization of lymphatic flow to optimize lymph node dissection.^100^, ^101^, ^102^

**FIGURE 4:**
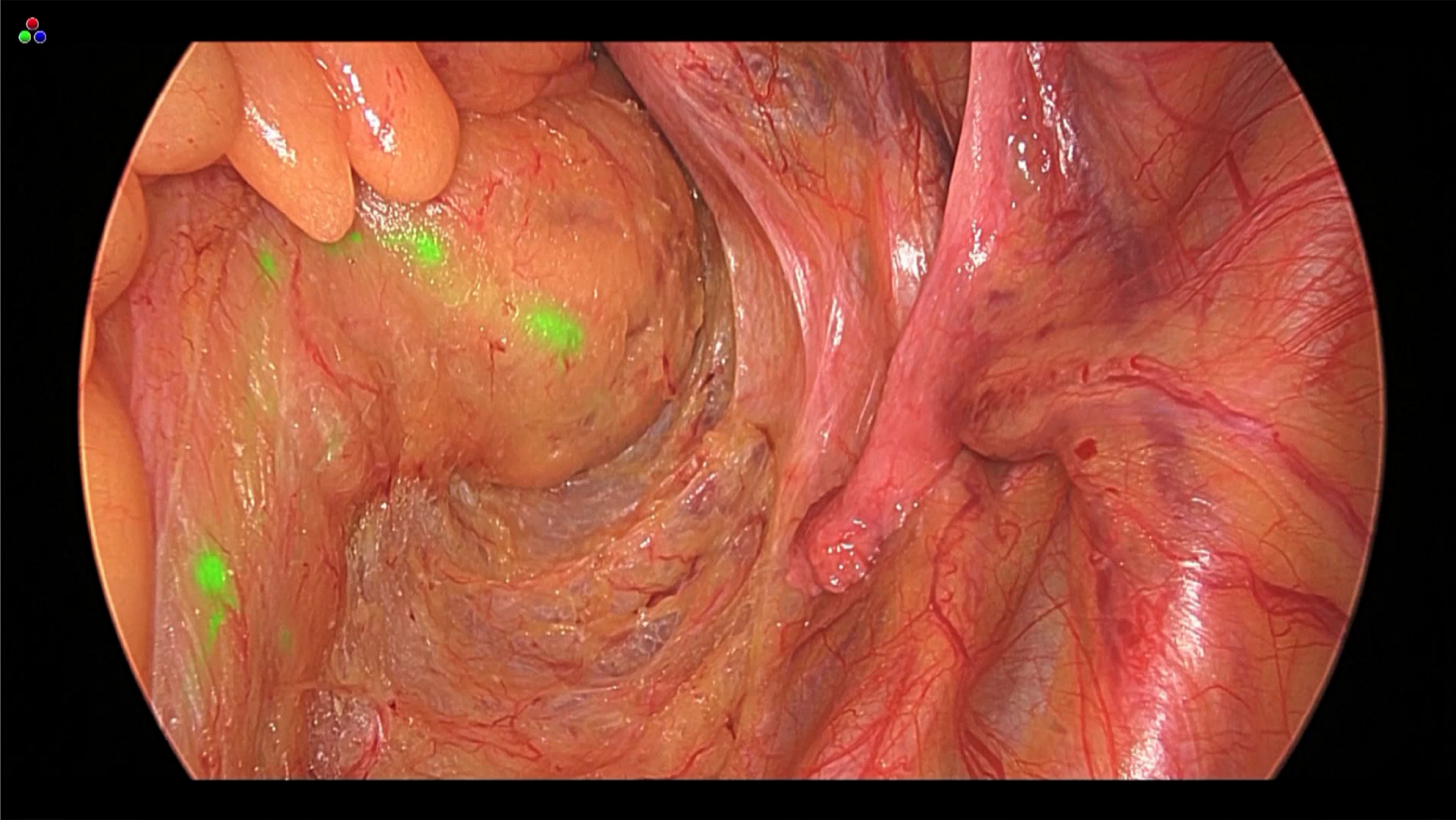
Fluorescence‐guided total mesorectal excision. Indocyanine green fluorescence overlaid on a white light image enables real‐time visualization of lymphatic flow. The mesorectal fascia enveloping the lymph flows and nodes as an intact package can be recognized during surgery for rectal cancer

We reported the usefulness of ICG fluorescence imaging instead of India ink tattooing for tumor site marking in laparoscopic surgery.^103^ The real‐time fluorescent lymphatic flow and lymph nodes can be detected with NIR light during surgery when ICG is injected into the submucosal layer (Figure 4). Fluorescence‐guided visualization of lymphatic flow might be useful for determining the extent of mesocolic excision and identifying the optimal dissection plane in colorectal cancer surgery. Determining the appropriate separation line of the mesentery is difficult during surgery due to blood vessel variation and complicated lymph flow between the hepatic and splenic flexure site. Watanabe et al demonstrated a lymph flow directed to the area of the root of the inferior mesenteric vein, which did not accompany the artery using ICG fluorescent imaging.^102^ In cases with lymph flow in various directions, the use of laparoscopic real‐time ICG fluorescent imaging can facilitate identifying the central vessels to be dissected and determine the appropriate line of the mesentery to be separated intraoperatively. Furthermore, fluorescence‐guided TME may help recognize the precise dissection plane along the mesorectal fascia enveloping the lymph flows and nodes as an intact package during surgery for rectal cancer, reducing the risk of local recurrence caused by disruption of the mesorectum. In laparoscopic LLND for middle‐lower rectal cancer, NIR fluorescence‐guided imaging could increase the total number of harvested lateral pelvic lymph nodes.^104^, ^105^ LLND in radical surgery incorporates complete removal of the region in which lymphatic flows from the lower rectum penetrate the pelvic plexus into the lateral space and ascend along the internal iliac arteries. NIR imaging is expected to confirm an absence of residual lymph nodes to be dissected. The quality of laparoscopic LLND can be improved by NIR with ICG.

Notably, ICG fluorescence imaging can assess lymph flow in real time only when the lymphatic vessel remains patent. Lymphatic vessel occlusion with tumor cells and bypassed lymph flow may occur in cases of LNM.^106^ The metastatic lymph nodes that are completely occupied by cancer do not fluoresce on ICG fluorescence imaging, regardless of the lymph node size.^107^ We investigated the association between metastasis and ICG fluorescence both in vivo and ex vivo to clarify whether ICG fluorescence imaging could be useful in colorectal cancer surgery. First, the ratio of metastatic lymph nodes among fluorescence‐negative lymph nodes was higher than among fluorescence‐positive nodes, and ICG fluorescence was not observed in metastatic lymph nodes with cancer cells occupying >90% of the total area. Second, the cancerous region in metastatic lymph nodes did not fluoresce under a fluorescence microscope.^108^ ICG fluorescence illustrates the lymph flow after the establishment of metastasis as a “snapshot” and may be helpful for NIR fluorescence‐guided surgery in colorectal cancer. The mesocolon, including the fluorescent lymph nodes, should be removed because metastatic lymph nodes occupied by cancer cells that do not fluoresce in real time during surgery are expected to be confined within the mesentery delineated by fluorescent lymph nodes.

### 4.2 Near‐infrared ureteral stent for preventing urethral injury

The urethra is difficult to detect during TaTME due to the view of the pelvic anatomy from the perineal side. Greater difficulty identifying the urethra in APE is a hurdle to applying the transanal approach.^109^ Misidentification of the urethra as the rectourethral muscle during dissection of the rectal anterior plane can result in urethral injury. An NIR spectroscopy ureteral stent, which was originally a medical device to fluorescently label the ureter, can also work as a marker to help identify the urethra in TaTME.^109^, ^110^ Such a novel device can be useful for implementing TaTME more safely.

## 5 MULTIMODAL THERAPY

### 5.1 Current standard of care: Neoadjuvant chemoradiotherapy

The Swedish Rectal Cancer Trial published in 1997 demonstrated that preoperative radiotherapy (RT) could prevent local recurrence and extend overall survival compared to surgery alone, showing the high curative effect of RT.^111^ Importantly, the patients in this trial were recruited from 1987 to 1990, when TME was not yet universally standardized. The rate of local recurrence in the Swedish trial was 23% for Dukes B and 40% for Dukes C, which is considerably higher than the current results. The Dutch trial recruited patients from 1996 to 1999 to investigate the effect of preoperative RT combined with TME, finding that preoperative RT could suppress local recurrence, as in the Swedish trial, but without a benefit to overall survival.^112^ Subsequent trials have shown that neoadjuvant CRT followed by surgery is the most efficacious strategy for local control,^113^, ^114^, ^115^, ^116^ leading to the establishment of neoadjuvant CRT as a standard of care for LARC. In consideration of the treatment strategy adopted in Western countries, Japanese guidelines in 2019 recommended neoadjuvant CRT for LARC at risk of local recurrence, but the indicated patients are not yet clearly defined.^117^

### 5.2 Challenges associated with neoadjuvant CRT

Unfortunately, most of the trials in the last 20 years did not extend disease‐free survival (DFS) or overall survival.^113^, ^114^, ^115^, ^116^ According to four recent trials comparing laparoscopic surgery and open surgery for LARC, the local recurrence rate declined to ~5%,^12^, ^13^, ^118^, ^119^ whereas the rate of distant metastasis remained at ~30% without evident improvement. How to address the high metastasis rate is currently the most important issue. A plausible explanation for the failure to achieve systemic control is that the compliance of patients completing both neoadjuvant therapy and surgery is suboptimal; therefore, adjuvant chemotherapy cannot be delivered sufficiently for the prevention of systemic relapse. To improve compliance for systemic chemotherapy, it has been postulated that chemotherapy can be administered before surgery at an increased dosage, which is the concept of total neoadjuvant therapy (TNT). The definition of TNT is combined neoadjuvant therapies using chemotherapy in conjunction with CRT/RT before surgery.^120^ TNT has a variety of treatment sequences (induction or consolidation chemotherapy) and duration of chemotherapy and irradiation methods (short or long course), and the most effective regimen is during the verification. To date, four RCTs have compared TNT and CRT, in which different TNTs were administered (Table 2). In the Polish II trial, TNT consisting of short‐course RT followed by three cycles of FOLFOX4 was compared to CRT in which CAPOX was used concomitantly.^121^ Although overall survival was superior to CRT at 3 y, other long‐term outcomes were not different, and the difference in overall survival disappeared at the 8‐y follow‐up.^122^ The STELLAR trial investigated the noninferiority of TNT to CRT by assessing DFS, demonstrating that the DFS was similar between the two treatments, although the overall survival of TNT was significantly better than that of CRT.^123^ In contrast, the RAPIDO and PRODIGE23 trials showed the better efficacy of TNT over CRT.^124^, ^125^ The short‐course RT followed by consolidation chemotherapy in RAPIDO and triplet induction chemotherapy followed by long‐course CRT in PRODIGE23 are different, although both treatments improved the long‐term results by mainly preventing distant metastasis. Based on the currently available results, we cannot conclude that TNT is more efficient than CRT, but it is likely that TNT would be beneficial and should be recommended for certain patients. The drawbacks of TNT should also be recognized adequately in order to safely introduce it into practice. TNT can be harmful due to overdosage, which can be related to the elevated risk of peripheral sensory neuropathy or other adverse events, or to the negative effects of irradiation, including urogenital dysfunction or the occurrence of secondary cancer. In Japan, where upfront TME with LLND has long been a standard of care, the results of TNT from Western countries cannot simply be extrapolated. To introduce TNT into future clinical practice, and how to identify appropriate patients is a critical challenge, and patient stratification to indicate TNT is one of the most fundamental issues (see Sections 6 and 7). The results of TNT have to be validated in a clinical trial, with careful introduction based on rigid judgment of indication.

**TABLE 2:** Summary of RCTs investigating TNT

Trial	Eligibility	Treatment arms	Number	pCR rate, %	DFS, %	LR, %	DM, %	OS, %
Polish II	Fixed cT3 or cT4	CRT (FOLFOX base)	254	12	52	21	27	65
		Short‐course RT followed by FOLFOX4 × 3 cycles	261	16	53	22	30	73
RAPIDO	cT4 or N2, EMVI+, MRF+	CRT	450	14	30.4^a^	6.0	26.8	88.8
		Short‐course RT followed by CAPOX × 6 or FOLFOX4 × 9 cycles	462	28	23.7^a^	8.3	20.0	89.1
PRODIGE23	cT3‐4 or N+	CRT	230	12	69	6	25	88
		FOLFIRINOX × 6 cycles followed by CRT	231	28	76	4	17	91
STELLAR	cT3‐4 or N+	CRT	297	12.3^b^	62.3	11	24.7	75.1
		Short‐course RT followed by CAPOX × 4 cycles	302	21.8^b^	64.5	8.4	22.8	86.5
OPRA	cStage II, III	Induction chemotherapy followed by CRT	158	N/A	76	6	16	N/A
		CRT followed by consolidation chemotherapy	166	N/A	76	6	18	N/A

^a^ Diseaserelated treatment failure.

^b^ pCR and sustained cCR.

Surgery has been an essential part of multimodal therapy and has a great therapeutic effect; however, it concurrently comprises surgical invasion, risk to sphincter preservation, possibility of stoma creation, or impaired urinary or sexual function for patients. The watch and wait (W&W) strategy has received attention in recent years, as once cCR is achieved, more than half of patients likely avoid local regrowth.^126^, ^127^, ^128^ It is possible that TNT can play a crucial role in the W&W strategy. Given that the local recurrence rate after surgery is comparable between TNT and CRT,^121^, ^122^, ^123^, ^124^, ^125^ we cannot argue that TNT is able to offer better local control, but TNT may improve the pCR rate according to a meta‐analysis, in which the odds of pCR are elevated ~40%.^120^ Conversely, the prognosis of non‐pCR cases is not necessarily favorable; therefore, it is also important not to assess the efficacy of TNT as a whole only with the pCR rate. Recently, the OPRA trial, which evaluated DFS as a primary endpoint between induction and consolidation chemotherapy with CRT, demonstrated that DFS is comparable between the two TNTs, whereas CRT followed by consolidation chemotherapy was associated with a higher rate of organ preservation.^129^ If we intend to offer the W&W approach for a patient, it may be possible that TNT including consolidation chemotherapy is more efficacious. It is also plausible that TNT may be effective for comparably early‐stage tumors, such as the “good” risk group defined in the ESMO guidelines, with an aim to achieve CR, but no evidence is currently available regarding whether this hypothesis is correct, which should be validated in future trials. The future treatment strategy for LARC may change dramatically.

### 5.3 Potential for omission of RT

In current multimodal therapy, RT is indispensable for local control. However, RT can have adverse events, including perineal wound infection, interference with surgical wound healing, genitourinary dysfunction, bowel obstruction, fractures, and increased risk of second cancer.^130^, ^131^, ^132^, ^133^ If local expansion of the tumor can be sufficiently controlled without RT, there may be a possibility to omit RT for such patients to avoid the adverse events. The FOWARC trial compared three treatment arms, including neoadjuvant CRT as standard of care, neoadjuvant CRT combined with FOLFOX6, and neoadjuvant FOLFOX6, demonstrating that long‐term outcomes were comparable among the three treatments, whereas treatment‐related adverse events were mitigated in neoadjuvant FOLFOX6.^134^, ^135^ This suggests that neoadjuvant chemotherapy (NAC) without RT can be an option for some LARCs. The ongoing PROSPECT trial (NCT01515787) is also enrolling patients for NAC. Several studies have evaluated the results of NAC in Japan, where the local control has been acceptable.^136^, ^137^, ^138^, ^139^, ^140^ LLND is a routinely performed procedure in Japan, and local control can be sufficiently feasible, suggesting that NAC is effective for systemic control and can play a crucial role in systematically eradicating cancer cells. Moreover, as a breakthrough, PD‐1 blockade by dostarlimab was shown to be effective for mismatch repair‐deficient, locally advanced rectal cancer.^141^ Surprisingly, pCR could be successfully achieved in all of the enrolled patients, indicating that ICI would also be an important part of multimodal therapy. In the future, the tumor indicated for omission of neoadjuvant RT may be clearly defined to promote precision medicine.

### 5.4 Future perspectives

Some patients can be offered the possibility of a W&W approach and can be cured without surgery. Nevertheless, surgery remains an important part of therapy for most patients who fail to achieve pCR or suffer from local regrowth. The indication of LLND in multimodal therapy should also be discussed, in which risk assessment for lateral LNM is mandatory.^59^, ^61^, ^142^, ^143^ Establishment of criteria for risk stratification is essential to guide optimal treatment for each patient, and molecular or imaging technology may serve efficiently.

## 6 MOLECULAR TECHNOLOGY FOR CANCER TREATMENT

Along with advances in surgical techniques and treatment methods, there have been advances in examination techniques to make appropriate treatment choices for individual patients. LNM is a strong prognostic factor in colorectal cancer, and postoperative adjuvant chemotherapy is recommended for patients with positive lymph nodes. The diagnosis of LNM is conventionally made pathologically by hematoxylin and eosin (HE) staining of the largest section of harvested lymph node, but LNM is judged to be absent when no cancer cells are seen on this section, even if small metastases to the lymph node have begun (false‐negative). One‐step nucleic acid amplification (OSNA) for cytokeratin 19 mRNA, a molecular assay, is a high‐sensitivity rapid assay for detection of micrometastases that was suggested as a potential predictor of recurrence risk, and clinical trials have demonstrated upstaging rates of 9.1% in pStage II colorectal cancer.^144^, ^145^ A large multicenter prospective study is currently under way to investigate the significance of the diagnosis of lymph node micrometastases by OSNA and the need for postoperative adjuvant chemotherapy in pStage II colorectal cancer. Furthermore, OSNA for perirectal lymph nodes may be useful for determining whether LLND should be performed due to its high negative predictive value.^146^ OSNA has the potential to attract attention as one of the factors for the stratification of patients with rectal cancer.

With the backdrop of technological innovations in next‐generation sequencing (NGS), liquid biopsy has emerged as a promising method to evaluate tumor‐related materials, including tumor cells, RNA, exosomes, and cell‐free DNA (cfDNA) in blood or other body fluids. Among these, cfDNA shed from tumor cells, called circulating tumor DNA (ctDNA), can now be detected in the circulation of patients with cancer. ctDNA can capture the real‐time tumor landscape, including the heterogeneity of multiple distinct subclones that may not be caught by tissue assessment, and may lead to the opportunity for additional treatment options.^147^ In addition, ctDNA has an extremely short half‐life in plasma (<2 h) compared to tumor markers, such as CEA and CA19‐9.^148^ Therefore, after curative resection of cancer, ctDNA is rapidly eliminated from the blood if there is no residual cancer, and a diagnostic system utilizing NGS technology has been developed to detect molecular residual disease (MRD), which is radiologically invisible, by taking advantage of this property of ctDNA.^149^, ^150^

Many studies have shown that MRD detection by ctDNA is promising for predicting recurrence. Tie et al found that, among 178 patients with stage II colon cancer not treated with adjuvant therapy, 11 of 14 (79%) with positive postoperative ctDNA and 16 of 164 (only 9.8%) with negative postoperative ctDNA recurred (hazard ratio [HR] = 18).^151^ Reinert et al also showed that the HR of relapse‐free survival was 7.2 in 94 stage II/III colorectal cancer patients who were ctDNA‐positive 30 d after surgery, and shortly after adjuvant chemotherapy, ctDNA‐positive patients were ~17 times (HR = 17.5) more likely to relapse than ctDNA‐negative patients.^152^ These data suggest that ctDNA is as an effective biomarker for stratifying patients at high risk of recurrence and that adjunction of ctDNA status into the TNM classification system may improve the prediction of colorectal cancer prognosis. The clinical usefulness of ctDNA for colorectal cancer is currently being assessed in a large‐scale platform named the CIRCULATE‐Japan project, which is composed of one observational study (GALAXY study) and two randomized phase III trials (VEGA and ALTAIR trials).^153^ The CIRCULATE‐Japan project uses Signatera (Natera, Austin, TX, USA), a novel, custom‐built ctDNA monitoring assay for MRD detection by a personalized blood test based on the unique signature of somatic single‐nucleotide variants identified in the individual's tumor. The GALAXY study, which began in 2020, is a large prospective nationwide registry to monitor ctDNA status in patients with clinical stage II to IV or recurrent colorectal cancer who can undergo complete surgical resection. Based on the results of the IDEA collaboration and the ACHIEVE trial, the 3‐y DFS for patients with high‐risk stage II and low‐risk stage III colon cancer treated with 3 mo of CAPOX is considered to be ~85% and, among these patients, the 3‐y DFS for those who are postoperatively ctDNA‐negative is expected to be ~89.5%.^154^, ^155^ The VEGA trial, a de‐escalation trial, is a randomized phase III study for postoperative ctDNA‐negative patients with high‐risk stage II and low‐risk stage III to evaluate whether surgery alone is noninferior to the standard CAPOX therapy (ie, whether omitting postoperative adjuvant chemotherapy may be acceptable). Although the VEGA trial is targeted at colon cancer, it has the potential to be applied to rectal cancer as well. The ctDNA‐positive group is a very high‐risk group for recurrence, and early intervention may be expected to prolong survival. The ALTAIR trial, an escalation trial, is a randomized, double‐blind, phase III study to evaluate the superiority of trifluridine/tipiracil (FTD/TPI) over placebo in colorectal cancer patients who are ctDNA‐positive at any time after curative surgery. A recent report showing the results of the DYNAMIC trial demonstrated that a ctDNA‐guided decision on adjuvant chemotherapy did not impair the prognosis and reduced adjuvant chemotherapy use,^156^ shedding light on the effect of ctDNA to optimize adjuvant therapy, although this hypothesis has to be demonstrated in ongoing interventional trials (ie, VEGA and ALTAIR).

Two new cohort studies were initiated in 2021 in the GALAXY study to further expand on preoperative ctDNA for stratification of colorectal cancer. One study is for pathological T1 (pT1) colorectal cancer and the other for rectal cancer with preoperative intervention. Current international guidelines recommend additional intestinal resection with lymph node dissection for high‐risk patients diagnosed with pT1 colorectal cancer who have undergone complete local resection but are at risk for LNM.^117^, ^157^ However, due to insufficient pathological criteria for risk stratification of LNMs, ~90% of patients without LNM are at risk of overtreatment that may impair their quality of life (QoL) due to the stoma or deterioration of defecation function, especially in rectal cancer patients. A new prospective study, DENEB, was launched for patients with pT1 colorectal cancer who underwent complete local resection and were scheduled for additional intestinal resection to investigate the predictive ability of LNM using ctDNA analysis compared to standard pathological criteria.^158^ As described above, the global standard treatment for locally advanced rectal cancer is neoadjuvant CRT followed by resection of the rectum, but there are currently no criteria for TNT. Considering adverse events and high medical costs, the application of TNT for all locally advanced rectal cancers can be excessive. Although preoperative ctDNA has been considered to have limited clinical relevance because it is not associated with progression or prognosis, preoperative the ctDNA results were recently shown to be strongly associated with subsequent distant metastatic recurrence when locally advanced rectal cancer is treated with neoadjuvant CRT.^159^, ^160^ Based on this finding, a new cohort was launched to optimize the indication for TNT using preoperative ctDNA after neoadjuvant CRT. The ctDNA assay is expected to provide new evidence for the establishment of a noninvasive personalized diagnosis and facilitate optimal treatment strategies for colorectal cancer patients.^161^

## 7 RECTAL CANCER DIAGNOSIS

In Western guidelines, MRI is regarded as an essential exam to precisely diagnose malignant features of rectal cancer.^2^, ^3^ The protocol for image acquisition is strictly defined, and the structured reporting system for MRI is systematized.^162^, ^163^ MRI can be used to evaluate T factor, subclassification of T3, invasion of anal sphincter, LNM, extramural vascular invasion (EMVI), and mucinous components, which are considered when deciding on individual treatment strategies. In the ESMO guidelines, several treatments are used, including TME first, neoadjuvant CRT, or TNT, depending on the MRI‐based tumor progression, wherein TNT is recommended for T4b or lateral lymph node‐positive cases.^2^ In the NCCN guidelines, TNT is recommended for T3–4 or lymph node‐positive cases.^3^

Different from Japan, Western countries have emphasized precise baseline diagnosis to guide appropriate neoadjuvant treatment, although a surgery‐first approach has long been used as the standard of care in Japan, which is partly reflected in the difference in attitudes toward MRI findings. In the MERCURY study, MRI‐involved CRM was verified to be the only risk factor for long‐term outcomes (not only local recurrence, but overall survival and DFS), which was a superior predictive marker to T or N factors.^164^ In the MERCURY II study, the risk factors for pathologically positive CRM were analyzed to decrease the positive CRM rate for rectal cancers lower than 6 cm from the anal verge.^165^ As a result, positive EMVI, MRI‐involved CRM, anterior tumor site, and tumor height lower than 4 cm were significant risk factors, which enabled the assessment of pCRM positivity. This preoperative assessment is useful to judge indications for neoadjuvant treatment.

Neoadjuvant CRT was introduced into the Japanese guidelines in 2019 as a treatment option. Thus, diagnosis of the tumor on MRI has become mandatory before determining treatment in Japan. Precise diagnosis is increasingly demanded, but this is not necessarily an easy task. The accuracy of MRI diagnosis has room for improvement, even in the data from the MERCURY group.^166^ In recent years, some attempts have been made to diagnose on MRI precisely. Artificial intelligence‐based technology has been constructed to visualize the rectal cancer area, which can potentially be utilized to find the malignant features of rectal cancer.^167^, ^168^, ^169^ In addition, according to a recent report, artificial intelligence can be utilized for preoperative simulation to visualize anatomical configuration individually, possibly improving operative safety.^170^ If the diagnostic accuracy could be more sophisticated, the treatment strategy may be optimized based on the status of the individual tumor, which would be beneficial for the patient.

## 8 LOW ANTERIOR RESECTION SYNDROME

Surgical treatment is shifting from uniform treatment to stratified and individualized treatment. The social need for not only curability and safety, but also preservation of function after cancer is high and one of the most important factors in cancer treatment. Functional impairments can occur in defecation, urination, and sexual function after rectal resection. Among these, bowel dysfunction after rectal resection used to be overlooked as an inevitable disorder after surgery. Recently, however, the term low anterior resection syndrome (LARS) has been used to describe “disordered bowel function after rectal resection, leading to a detriment in QoL,”^171^ and the diagnosis, treatment, and prevention of these disorders have been the focus of much attention. However, LARS is a concept that encompasses a variety of symptoms, from fecal incontinence and urgency, frequent stools, and clustering to evacuation difficulties, and its complex symptoms and pathology have long prevented the establishment of a diagnosis or definition. Thus, reports on LARS have been variable, using the Wexner score for fecal incontinence or assessing only individual symptoms.^172^ Therefore, meta‐analyses and systemic reviews were not available, and even the prevalence of LARS was not clear.^173^

The LARS score was developed by Emmertsen and Laurberg in 2012 as a scoring system that can easily evaluate multiple symptoms of LARS according to the level of QoL impairment.^174^ The LARS score highly correlates with the EORTC QLQ‐C30,^175^, ^176^ CR38,^177^ and SF36,^178^ and its clinical usefulness has been described in several articles. The LARS score has been translated into more than 30 languages, and we reported the validation of the Japanese version of the LARS score through double translation in 2018.^179^ The LARS score enables easy diagnosis and evaluation of LARS; furthermore, international comparison is possible by using the score created and validated in accordance with international standards.

Assessment of the diagnosis and severity of LARS based on certain criteria has also revealed risk factors for severe LARS. Previously, low rectal tumor, TME,^180^ prior radiation therapy,^181^ and age^182^ were described as factors. Recent advances in surgical techniques have increased the number of cases of super low anterior resection and intersphincteric resection for low‐lying rectal tumors, and the number of high‐risk cases of LARS has increased.^183^ However, no consensus has been reached on whether TaTME is a risk factor for LARS.^38^ In addition, as the number of surgical cases after RT increases with the spread of multimodal therapy, high‐risk cases of severe LARS are expected to increase, and treatment and prevention of LARS will become increasingly more important for selecting the best treatment for patients.

Despite LARS being a serious complication that impairs patient QoL, interventions and treatment for LARS vary widely among institutions. LARS intervention begins with preoperative risk assessment and appropriate informed consent, followed by a multifaceted approach after LARS onset, including lifestyle and dietary guidance, drug therapy, and pelvic floor muscle rehabilitation with biofeedback therapy, transanal irrigation, and sacral nerve stimulation.^184^ Cooperation between physicians, nurses, and other professionals is also important in the treatment of LARS. When performing rectal cancer surgery, surgeons should care not only for the cure of the cancer, but also for the prevention and treatment of LARS.

In 2020, the LARS International collaborative group, including the authors of the LARS score, announced a proposal for a new definition of LARS including eight symptoms of defecation disorder and eight items affecting QoL.^185^ A new scoring system based on the definition of LARS for follow‐up and evaluation of treatment efficacy will be presented soon. The LARS score and the new score are anticipated to enable the sharing of detailed information internationally, and research leading to the treatment and prevention of LARS is expected in the future.

## 9 UROGENITAL DYSFUNCTION

Urinary dysfunction occurs after TME at a fixed frequency of 30%–50%,^186^, ^187^ which may cause deterioration of the patient's QoL or induce urinary tract infection. This postoperative complication is mainly due to disturbance of the nervous system, including the pelvic plexus and neurovascular bundle.^188^, ^189^ The addition of LLND to TME can reportedly increase the risk more, at an odds ratio of about 2,^190^, ^191^ but JCOG0212 demonstrated that a difference in urinary dysfunction could not be found between TME alone and TME with nerve‐preserving LLND.^187^ These data suggest that difficult manipulation in the deep pelvis can result in inadvertent injury to surrounding autonomic nerves. Being male, having a low level of anastomosis, anterior tumor, or increased blood loss have been demonstrated to be risk factors for urinary dysfunction after TME in previous studies, all of which are possibly related to an elevated risk of nerve injury.^187^, ^188^, ^192^, ^193^ Sexual dysfunction is also likely to be compromised after rectal cancer resection in more than half of patients, although the definition of sexual dysfunction is not standardized.^186^ This result is caused by multifactorial effects, including nerve injury and preoperative radiotherapy.^194^

In recent years, the above‐mentioned evolution in minimally invasive surgery has dramatically improved the visibility or maneuverability in the deep pelvis, and robot‐assisted laparoscopic surgery would be efficacious for preserving autonomic nerves.^195^, ^196^, ^197^ A meta‐analysis investigating the effect on urinary and sexual dysfunction demonstrated that the function after robotic approach was favorable compared to the laparoscopic approach, especially in male patients.^27^ In taking this evidence into consideration, robot‐assisted laparoscopic surgery can be beneficial in terms of postoperative urinary and sexual dysfunction, but this should be validated in a prospective study. The current VITRUVIANO trial is investigating CRM, as well as urinary dysfunction, and we are going to demonstrate the significance of its use in light of the urinary function.

## 10 SUMMARY

In this review, we summarized various perspectives regarding treatment, diagnosis, molecular technology, enhanced visualization, and postoperative function in rectal cancer. Now that we are faced with a paradigm shift toward improved rectal cancer treatment, it is necessary to be familiar with these details comprehensively to offer optimized treatment for patients. We have to consider the indication of multimodal therapy based on accurate diagnosis, in which MRI is inevitable. In planning the surgical approach, we have to take measures to achieve complete TME with sufficient CRM or discuss with the patient whether they desire anal preservation. A recent concept, the W&W approach, may also be suggested for certain patients depending on the response after neoadjuvant therapy. The W&W approach should be offered by sufficiently considering the risk–benefit balance compared to the Japanese results. Such dramatic changes in the treatment of rectal cancer demand an understanding of the efficacy of each modality in providing precision treatment.

## CONFLICT OF INTEREST

The other authors declare no conflicts of interest. Ichiro Takemasa is the editorial member of the *Annals of Gastroenterologial Surgery*.

## References

[1] Sung H , Ferlay J , Siegel RL , Laversanne M , Soerjomataram I , Jemal A , et al. Global cancer statistics 2020: GLOBOCAN estimates of incidence and mortality worldwide for 36 cancers in 185 countries. CA Cancer J Clin. 2021;71(3):209–49. 33538338 10.3322/caac.21660

[2] Glynne‐Jones R , Wyrwicz L , Tiret E , Brown G , Rödel C , Cervantes A , et al. Rectal cancer: ESMO clinical practice guidelines for diagnosis, treatment and follow‐up†. Ann Oncol. 2017;28(suppl_4):iv22–40. 28881920 10.1093/annonc/mdx224

[3] Benson A. NCCN guideline<rectal‐2.pdf>. 2022.

[4] Colon Cancer Laparoscopic or Open Resection Study G , Buunen M , Veldkamp R , et al. Survival after laparoscopic surgery versus open surgery for colon cancer: long‐term outcome of a randomised clinical trial. Lancet Oncol. 2009;10(1):44–52. 19071061 10.1016/S1470-2045(08)70310-3

[5] Guillou PJ , Quirke P , Thorpe H , Walker J , Jayne DG , Smith AMH , et al. Short‐term endpoints of conventional versus laparoscopic‐assisted surgery in patients with colorectal cancer (MRC CLASICC trial): multicentre, randomised controlled trial. Lancet. 2005;365(9472):1718–26. 15894098 10.1016/S0140-6736(05)66545-2

[6] Jayne DG , Thorpe HC , Copeland J , Quirke P , Brown JM , Guillou PJ . Five‐year follow‐up of the Medical Research Council CLASICC trial of laparoscopically assisted versus open surgery for colorectal cancer. Br J Surg. 2010;97(11):1638–45. 20629110 10.1002/bjs.7160

[7] Kitano S , Inomata M , Mizusawa J , Katayama H , Watanabe M , Yamamoto S , et al. Survival outcomes following laparoscopic versus open D3 dissection for stage II or III colon cancer (JCOG0404): a phase 3, randomised controlled trial. Lancet Gastroenterol Hepatol. 2017;2(4):261–8. 28404155 10.1016/S2468-1253(16)30207-2

[8] Yamamoto S , Inomata M , Katayama H , Mizusawa J , Etoh T , Konishi F , et al. Short‐term surgical outcomes from a randomized controlled trial to evaluate laparoscopic and open D3 dissection for stage II/III colon cancer: Japan clinical oncology Group study JCOG 0404. Ann Surg. 2014;260(1):23–30. 24509190 10.1097/SLA.0000000000000499

[9] Yamamoto S , Ito M , Okuda J , Fujii S , Yamaguchi S , Yoshimura K , et al. Laparoscopic surgery for stage 0/I rectal carcinoma: short‐term outcomes of a single‐arm phase II trial. Ann Surg. 2013;258(2):283–8. 23426337 10.1097/SLA.0b013e318283669c

[10] Marubashi S , Takahashi A , Kakeji Y , Hasegawa H , Ueno H , Eguchi S , et al. Surgical outcomes in gastroenterological surgery in Japan: report of the National Clinical Database 2011‐2019. Ann Gastroenterol Surg. 2021;5(5):639–58. 34585049 10.1002/ags3.12462 PMC8452469

[11] Sun Z , Kim J , Adam MA , Nussbaum DP , Speicher PJ , Mantyh CR , et al. Minimally invasive versus open low anterior resection: equivalent survival in a National Analysis of 14,033 patients with rectal cancer. Ann Surg. 2016;263(6):1152–8. 26501702 10.1097/SLA.0000000000001388

[12] Bonjer HJ , Deijen CL , Abis GA , Cuesta MA , van der Pas MHGM , de Lange‐de Klerk ESM , et al. A randomized trial of laparoscopic versus open surgery for rectal cancer. N Engl J Med. 2015;372(14):1324–32. 25830422 10.1056/NEJMoa1414882

[13] Jeong SY , Park JW , Nam BH , Kim S , Kang SB , Lim SB , et al. Open versus laparoscopic surgery for mid‐rectal or low‐rectal cancer after neoadjuvant chemoradiotherapy (COREAN trial): survival outcomes of an open‐label, noninferiority, randomised controlled trial. Lancet Oncol. 2014;15(7):767–74. 24837215 10.1016/S1470-2045(14)70205-0

[14] Fleshman J , Branda M , Sargent DJ , Boller AM , George V , Abbas M , et al. Effect of laparoscopic‐assisted resection vs open resection of stage II or III rectal cancer on pathologic outcomes: the ACOSOG Z6051 randomized clinical trial. JAMA. 2015;314(13):1346–55. 26441179 10.1001/jama.2015.10529 PMC5140087

[15] Stevenson AR , Solomon MJ , Lumley JW , et al. Effect of laparoscopic‐assisted resection vs open resection on pathological outcomes in rectal cancer: the ALaCaRT randomized clinical trial. JAMA. 2015;314(13):1356–63. 26441180 10.1001/jama.2015.12009

[16] Hiraki M , Takemasa I , Uemura M , Haraguchi N , Nishimura J , Hata T , et al. Evaluation of invasiveness in single‐site laparoscopic colectomy, using "the PainVision™ system" for quantitative analysis of pain sensation. Surg Endosc. 2014;28(11):3216–23. 25027469 10.1007/s00464-014-3594-7

[17] Hamabe A , Takemasa I , Hata T , Mizushima T , Doki Y , Mori M . Patient body image and satisfaction with surgical wound appearance after reduced port surgery for colorectal diseases. World J Surg. 2016;40(7):1748–54. 27094561 10.1007/s00268-016-3414-4

[18] Miyo M , Takemasa I , Ishihara H , Hata T , Mizushima T , Ohno Y , et al. Long‐term outcomes of single‐site laparoscopic colectomy with complete Mesocolic excision for colon cancer: comparison with conventional multiport laparoscopic colectomy using propensity score matching. Dis Colon Rectum. 2017;60(7):664–73. 28594715 10.1097/DCR.0000000000000810

[19] Takemasa I , Uemura M , Nishimura J , Mizushima T , Yamamoto H , Ikeda M , et al. Feasibility of single‐site laparoscopic colectomy with complete mesocolic excision for colon cancer: a prospective case‐control comparison. Surg Endosc. 2014;28(4):1110–8. 24202709 10.1007/s00464-013-3284-x PMC3973946

[20] Gómez Ruiz M , Lainez Escribano M , Cagigas Fernández C , Cristobal Poch L , Santarrufina MS . Robotic surgery for colorectal cancer. Ann Gastroenterol Surg. 2020;4(6):646–51. 33319154 10.1002/ags3.12401 PMC7726686

[21] Matsuyama T , Kinugasa Y , Nakajima Y , Kojima K . Robotic‐assisted surgery for rectal cancer: current state and future perspective. Ann Gastroenterol Surg. 2018;2(6):406–12. 30460343 10.1002/ags3.12202 PMC6236106

[22] Jayne D , Pigazzi A , Marshall H , Croft J , Corrigan N , Copeland J , et al. Effect of robotic‐assisted vs conventional laparoscopic surgery on risk of conversion to open laparotomy among patients undergoing resection for rectal cancer: the ROLARR randomized clinical trial. JAMA. 2017;318(16):1569–80. 29067426 10.1001/jama.2017.7219 PMC5818805

[23] Matsuyama T , Endo H , Yamamoto H , Takemasa I , Uehara K , Hanai T , et al. Outcomes of robot‐assisted versus conventional laparoscopic low anterior resection in patients with rectal cancer: propensity‐matched analysis of the National Clinical Database in Japan. BJS Open. 2021;5(5):zrab083. 34553225 10.1093/bjsopen/zrab083 PMC8458638

[24] Feng Q , Yuan W , Li T , Tang B , Jia B , Zhou Y , et al. Robotic versus laparoscopic surgery for middle and low rectal cancer (REAL): short‐term outcomes of a multicentre randomised controlled trial. Lancet Gastroenterol Hepatol. 2022;7(11):991–1004. 36087608 10.1016/S2468-1253(22)00248-5

[25] Qiu H , Yu D , Ye S , Shan R , Ai J , Shi J . Long‐term oncological outcomes in robotic versus laparoscopic approach for rectal cancer: a systematic review and meta‐analysis. Int J Surg. 2020;80:225–30. 32251720 10.1016/j.ijsu.2020.03.009

[26] Leitao MM Jr , Kreaden US , Laudone V , et al. The RECOURSE study: long‐term oncologic outcomes associated with robotically assisted minimally invasive procedures for endometrial, cervical, cOlorectal, lUng, or pRoState cancEr: a systematic review and meta‐analysis. Ann Surg. 2022. 10.1097/SLA.0000000000005698. Online ahead of print. PMC9905254 36073772

[27] Fleming CA , Cullinane C , Lynch N , Killeen S , Coffey JC , Peirce CB . Urogenital function following robotic and laparoscopic rectal cancer surgery: meta‐analysis. Br J Surg. 2021;108(2):128–37. 33711141 10.1093/bjs/znaa067

[28] Heald RJ , Husband EM , Ryall RD . The mesorectum in rectal cancer surgery‐‐the clue to pelvic recurrence? Br J Surg. 1982;69(10):613–6. 6751457 10.1002/bjs.1800691019

[29] Adam IJ , Mohamdee MO , Martin IG , et al. Role of circumferential margin involvement in the local recurrence of rectal cancer. Lancet. 1994;344(8924):707–11. 7915774 10.1016/s0140-6736(94)92206-3

[30] Ishii M , Takemasa I , Okita K , Okuya K , Hamabe A , Nishidate T , et al. A modified method for resected specimen processing in rectal cancer: semi‐opened with transverse slicing for measuring of the circumferential resection margin. Asian J Endosc Surg. 2022;15(2):437–42. 34743420 10.1111/ases.13003

[31] Takemasa I , Okuya K , Okita K , Ishii M , Ito M , Uehara K , et al. Feasibility of the semi‐opened method of specimen resection for a circumferential resection margin in rectal cancer surgery: a multicenter study. Surg Today. 2022;52:1275–83. 35378663 10.1007/s00595-022-02481-z

[32] Takemasa I , Hamabe A , Ito M , et al. Japanese multicenter prospective study investigating laparoscopic surgery for locally advanced rectal cancer with evaluation of CRM and TME quality (PRODUCT trial). Annals Gastroenterol Surg. 2022;6:767–77. 10.1002/ags3.12592 PMC9628237 36338586

[33] Sylla P , Rattner DW , Delgado S , Lacy AM . NOTES transanal rectal cancer resection using transanal endoscopic microsurgery and laparoscopic assistance. Surg Endosc. 2010;24(5):1205–10. 20186432 10.1007/s00464-010-0965-6

[34] de Lacy AM , Rattner DW , Adelsdorfer C , Tasende MM , Fernández M , Delgado S , et al. Transanal natural orifice transluminal endoscopic surgery (NOTES) rectal resection: "down‐to‐up" total mesorectal excision (TME)‐‐short‐term outcomes in the first 20 cases. Surg Endosc. 2013;27(9):3165–72. 23519489 10.1007/s00464-013-2872-0

[35] Denost Q , Adam JP , Rullier A , Buscail E , Laurent C , Rullier E . Perineal transanal approach: a new standard for laparoscopic sphincter‐saving resection in low rectal cancer, a randomized trial. Ann Surg. 2014;260(6):993–9. 24950270 10.1097/SLA.0000000000000766

[36] Velthuis S , Nieuwenhuis DH , Ruijter TE , Cuesta MA , Bonjer HJ , Sietses C . Transanal versus traditional laparoscopic total mesorectal excision for rectal carcinoma. Surg Endosc. 2014;28(12):3494–9. 24972923 10.1007/s00464-014-3636-1

[37] Fernandez‐Hevia M , Delgado S , Castells A , et al. Transanal total mesorectal excision in rectal cancer: short‐term outcomes in comparison with laparoscopic surgery. Ann Surg. 2015;261(2):221–7. 25185463 10.1097/SLA.0000000000000865

[38] Hajibandeh S , Hajibandeh S , Eltair M , George AT , Thumbe V , Torrance AW , et al. Meta‐analysis of transanal total mesorectal excision versus laparoscopic total mesorectal excision in management of rectal cancer. Int J Colorectal Dis. 2020;35(4):575–93. 32124047 10.1007/s00384-020-03545-7

[39] Roodbeen SX , Spinelli A , Bemelman WA , et al. Local recurrence after Transanal Total Mesorectal excision for rectal cancer: a multicenter cohort study. Ann Surg. 2020;274:359–66. 10.1097/SLA.0000000000003757 31972648

[40] Yamamoto S . Comparison of the perioperative outcomes of laparoscopic surgery, robotic surgery, open surgery, and transanal total mesorectal excision for rectal cancer: an overview of systematic reviews. Ann Gastroenterol Surg. 2020;4(6):628–34. 33319152 10.1002/ags3.12385 PMC7726682

[41] Simillis C , Lal N , Thoukididou SN , Kontovounisios C , Smith JJ , Hompes R , et al. Open versus laparoscopic versus robotic versus Transanal Mesorectal excision for rectal cancer: a systematic review and network meta‐analysis. Ann Surg. 2019;270(1):59–68. 30720507 10.1097/SLA.0000000000003227

[42] Hamabe A , Okita K , Nishidate T , Okuya K , Akizuki E , Sato Y , et al. Transperineal minimally invasive abdominoperineal excision for rectal cancer based on anatomical analysis of the muscular structure. Asian J Endosc Surg. 2021;14:675–83. 33561899 10.1111/ases.12917

[43] van Oostendorp SE , Roodbeen SX , Chen CC , Caycedo‐Marulanda A , Joshi HM , Tanis PJ , et al. Transperineal minimally invasive APE: preliminary outcomes in a multicenter cohort. Tech Coloproctol. 2020;24(8):823–31. 32556867 10.1007/s10151-020-02234-5 PMC7359144

[44] Hamabe A , Okita K , Nishidate T , Okuya K , Akizuki E , Sato Y , et al. Short‐term outcomes with standardized Transperineal minimally invasive abdominoperineal excision for rectal cancer. J Gastrointest Surg. 2022;26(3):713–9. 34608600 10.1007/s11605-021-05140-9

[45] Penna M , Hompes R , Arnold S , Wynn G , Austin R , Warusavitarne J , et al. Incidence and risk factors for anastomotic failure in 1594 patients treated by Transanal Total Mesorectal excision: results from the international TaTME registry. Ann Surg. 2019;269(4):700–11. 29315090 10.1097/SLA.0000000000002653

[46] Sylla P , Knol JJ , D'Andrea AP , et al. Urethral injury and other urologic injuries during Transanal Total Mesorectal excision: an international collaborative study. Ann Surg. 2019;274:e115–25. 10.1097/SLA.0000000000003597 31567502

[47] Wasmuth HH , Faerden AE , Myklebust TA , Pfeffer F , Norderval S , Riis R , et al. Transanal total mesorectal excision for rectal cancer has been suspended in Norway. Br J Surg. 2020;107(1):121–30. 31802481 10.1002/bjs.11459

[48] van Oostendorp SE , Belgers HJ , Bootsma BT , et al. Locoregional recurrences after transanal total mesorectal excision of rectal cancer during implementation. Br J Surg. 2020;107(9):1211–20. 32246472 10.1002/bjs.11525 PMC7496604

[49] Hol JC , van Oostendorp SE , Tuynman JB , Sietses C . Long‐term oncological results after transanal total mesorectal excision for rectal carcinoma. Tech Coloproctol. 2019;23(9):903–11. 31599385 10.1007/s10151-019-02094-8 PMC6791915

[50] Gonzalez‐Abos C , de Lacy FB , Guzman Y , et al. Transanal total mesorectal excision for stage II or III rectal cancer: pattern of local recurrence in a tertiary referral center. Surg Endosc. 2021;35(12):7191–9. 33398553 10.1007/s00464-020-08200-4

[51] Hompes R , Reply to Gachabayov the TaTME Guidance Group representing the ESCP (European Society of Coloproctology), in collaboration with ASCRS (American Society of Colon, Rectum Surgeons), ACPGBI (Association of Coloproctology of Great Britain and Ireland), ECCO (European Crohn's, Colitis Organisation), EAES (European Association of Endoscopic Surgeons), ESSO (European Society of Surgical Oncology), CSCRS (Canadian Society of Colorectal Surgery), CNSCRS (Chinese Society of Colorectal Surgery), CSLES (Chinese Society of Laparo‐Endoscopi) , Adamina M , Aigner F , Araujo S , Arezzo A , et al. Consensus statement on TaTME: other thoughts. Colorectal Dis. 2021;23(2):553–5.33170997

[52] Francis N , Penna M , Mackenzie H , Carter F , Hompes R , International Ta TMEECG . Consensus on structured training curriculum for transanal total mesorectal excision (TaTME). Surg Endosc. 2017;31(7):2711–9. 28462478 10.1007/s00464-017-5562-5

[53] Deijen CL , Velthuis S , Tsai A , Mavroveli S , de Lange‐de Klerk ESM , Sietses C , et al. COLOR III: a multicentre randomised clinical trial comparing transanal TME versus laparoscopic TME for mid and low rectal cancer. Surg Endosc. 2016;30(8):3210–5. 26537907 10.1007/s00464-015-4615-x PMC4956704

[54] Lelong B , de Chaisemartin C , Meillat H , et al. A multicentre randomised controlled trial to evaluate the efficacy, morbidity and functional outcome of endoscopic transanal proctectomy versus laparoscopic proctectomy for low‐lying rectal cancer (ETAP‐GRECCAR 11 TRIAL): rationale and design. BMC Cancer. 2017;17(1):253. 28399840 10.1186/s12885-017-3200-1 PMC5387204

[55] Sugihara K , Kobayashi H , Kato T , Mori T , Mochizuki H , Kameoka S , et al. Indication and benefit of pelvic sidewall dissection for rectal cancer. Dis Colon Rectum. 2006;49(11):1663–72. 17041749 10.1007/s10350-006-0714-z

[56] Kusters M , Beets GL , van de Velde CJ , et al. A comparison between the treatment of low rectal cancer in Japan and The Netherlands, focusing on the patterns of local recurrence. Ann Surg. 2009;249(2):229–35. 19212175 10.1097/SLA.0b013e318190a664

[57] Fujita S , Mizusawa J , Kanemitsu Y , Ito M , Kinugasa Y , Komori K , et al. Mesorectal excision with or without lateral lymph node dissection for clinical stage II/III lower rectal cancer (JCOG0212): a multicenter, randomized controlled, noninferiority trial. Ann Surg. 2017;266(2):201–7. 28288057 10.1097/SLA.0000000000002212

[58] Akiyoshi T , Matsueda K , Hiratsuka M , et al. Indications for lateral pelvic lymph node dissection based on magnetic resonance imaging before and after preoperative Chemoradiotherapy in patients with advanced low‐rectal cancer. Ann Surg Oncol. 2015;22(Suppl 3):S614–20. 25896145 10.1245/s10434-015-4565-5

[59] Ogura A , Konishi T , Cunningham C , Garcia‐Aguilar J , Iversen H , Toda S , et al. Neoadjuvant (chemo)radiotherapy with Total Mesorectal excision only is not sufficient to prevent lateral local recurrence in enlarged nodes: results of the multicenter lateral node study of patients with low cT3/4 rectal cancer. J Clin Oncol. 2019;37(1):33–43. 30403572 10.1200/JCO.18.00032 PMC6366816

[60] Takemasa I . Advances and controversies in treatment for locally advanced rectal cancer over the past decades: west meets east. Ann Gastroenterol Surg. 2020;4(4):314–5. 32724873 10.1002/ags3.12371 PMC7382423

[61] Hamabe A , Ishii M , Onodera K , Okita K , Nishidate T , Okuya K , et al. MRI‐detected extramural vascular invasion potentiates the risk for pathological metastasis to the lateral lymph nodes in rectal cancer. Surg Today. 2021;51(10):1583–93. 33665727 10.1007/s00595-021-02250-4

[62] Sumii A , Hida K , Sakai Y , Hoshino N , Nishizaki D , Akagi T , et al. Establishment and validation of a nomogram for predicting potential lateral pelvic lymph node metastasis in low rectal cancer. Int J Clin Oncol. 2022;27:1173–9. 35415787 10.1007/s10147-022-02157-1

[63] Smith NJ , Barbachano Y , Norman AR , Swift RI , Abulafi AM , Brown G . Prognostic significance of magnetic resonance imaging‐detected extramural vascular invasion in rectal cancer. Br J Surg. 2008;95(2):229–36. 17932879 10.1002/bjs.5917

[64] Furuhata T , Okita K , Nishidate T , Ito T , Yamaguchi H , Ueki T , et al. Clinical feasibility of laparoscopic lateral pelvic lymph node dissection following total mesorectal excision for advanced rectal cancer. Surg Today. 2015;45(3):310–4. 24792010 10.1007/s00595-014-0906-4

[65] Ogura A , Akiyoshi T , Nagasaki T , Konishi T , Fujimoto Y , Nagayama S , et al. Feasibility of laparoscopic Total Mesorectal excision with extended lateral pelvic lymph node dissection for advanced lower rectal cancer after preoperative Chemoradiotherapy. World J Surg. 2017;41(3):868–75. 27730352 10.1007/s00268-016-3762-0

[66] Yamaguchi T , Kinugasa Y , Shiomi A , Tomioka H , Kagawa H . Robotic‐assisted laparoscopic versus open lateral lymph node dissection for advanced lower rectal cancer. Surg Endosc. 2016;30(2):721–8. 26092002 10.1007/s00464-015-4266-y

[67] Kim HJ , Choi GS , Park JS , Park SY , Lee HJ , Woo IT , et al. Selective lateral pelvic lymph node dissection: a comparative study of the robotic versus laparoscopic approach. Surg Endosc. 2018;32(5):2466–73. 29124406 10.1007/s00464-017-5948-4

[68] Yamaguchi T , Kinugasa Y , Shiomi A , Kagawa H , Yamakawa Y , Furutani A , et al. Oncological outcomes of robotic‐assisted laparoscopic versus open lateral lymph node dissection for locally advanced low rectal cancer. Surg Endosc. 2018;32(11):4498–505. 29721748 10.1007/s00464-018-6197-x

[69] Kanemitsu Y , Komori K , Shida D , Ochiai H , Tsukamoto S , Kinoshita T , et al. Potential impact of lateral lymph node dissection (LLND) for low rectal cancer on prognoses and local control: a comparison of 2 high‐volume centers in Japan that employ different policies concerning LLND. Surgery. 2017;162(2):303–14. 28366499 10.1016/j.surg.2017.02.005

[70] Mirnezami A , Mirnezami R , Chandrakumaran K , Sasapu K , Sagar P , Finan P . Increased local recurrence and reduced survival from colorectal cancer following anastomotic leak: systematic review and meta‐analysis. Ann Surg. 2011;253(5):890–9. 21394013 10.1097/SLA.0b013e3182128929

[71] Krarup PM , Nordholm‐Carstensen A , Jorgensen LN , Harling H . Anastomotic leak increases distant recurrence and long‐term mortality after curative resection for colonic cancer: a nationwide cohort study. Ann Surg. 2014;259(5):930–8. 24045445 10.1097/SLA.0b013e3182a6f2fc

[72] Kang J , Choi GS , Oh JH , Kim NK , Park JS , Kim MJ , et al. Multicenter analysis of long‐term oncologic impact of anastomotic leakage after laparoscopic Total Mesorectal excision: the Korean laparoscopic colorectal surgery study Group. Medicine (Baltimore). 2015;94(29):e1202. 26200636 10.1097/MD.0000000000001202 PMC4603022

[73] Nakanishi R , Fujimoto Y , Sugiyama M , Hisamatsu Y , Nakanoko T , Ando K , et al. Clinical impact of the triple‐layered circular stapler for reducing the anastomotic leakage in rectal cancer surgery: porcine model and multicenter retrospective cohort analysis. Ann Gastroenterol Surg. 2022;6(2):256–64. 35261951 10.1002/ags3.12516 PMC8889859

[74] Kologlu M , Yorganci K , Renda N , Sayek I . Effect of local and remote ischemia‐reperfusion injury on healing of colonic anastomoses. Surgery. 2000;128(1):99–104. 10876192 10.1067/msy.2000.107414

[75] McDermott FD , Heeney A , Kelly ME , Steele RJ , Carlson GL , Winter DC . Systematic review of preoperative, intraoperative and postoperative risk factors for colorectal anastomotic leaks. Br J Surg. 2015;102(5):462–79. 25703524 10.1002/bjs.9697

[76] Chadi SA , Fingerhut A , Berho M , DeMeester SR , Fleshman JW , Hyman NH , et al. Emerging trends in the etiology, prevention, and treatment of gastrointestinal anastomotic leakage. J Gastrointest Surg. 2016;20(12):2035–51. 27638764 10.1007/s11605-016-3255-3

[77] Karliczek A , Harlaar NJ , Zeebregts CJ , Wiggers T , Baas PC , van Dam GM . Surgeons lack predictive accuracy for anastomotic leakage in gastrointestinal surgery. Int J Colorectal Dis. 2009;24(5):569–76. 19221768 10.1007/s00384-009-0658-6

[78] Jafari MD , Wexner SD , Martz JE , McLemore EC , Margolin DA , Sherwinter DA , et al. Perfusion assessment in laparoscopic left‐sided/anterior resection (PILLAR II): a multi‐institutional study. J Am Coll Surg. 2015;220(1):82–92 e81. 25451666 10.1016/j.jamcollsurg.2014.09.015

[79] Watanabe J , Ota M , Suwa Y , Suzuki S , Suwa H , Momiyama M , et al. Evaluation of the intestinal blood flow near the rectosigmoid junction using the indocyanine green fluorescence method in a colorectal cancer surgery. Int J Colorectal Dis. 2015;30(3):329–35. 25598047 10.1007/s00384-015-2129-6

[80] Boni L , Fingerhut A , Marzorati A , Rausei S , Dionigi G , Cassinotti E . Indocyanine green fluorescence angiography during laparoscopic low anterior resection: results of a case‐matched study. Surg Endosc. 2017;31(4):1836–40. 27553790 10.1007/s00464-016-5181-6

[81] Mizrahi I , Abu‐Gazala M , Rickles AS , Fernandez LM , Petrucci A , Wolf J , et al. Indocyanine green fluorescence angiography during low anterior resection for low rectal cancer: results of a comparative cohort study. Tech Coloproctol. 2018;22(7):535–40. 30097803 10.1007/s10151-018-1832-z

[82] Ishii M , Hamabe A , Okita K , Nishidate T , Okuya K , Usui A , et al. Efficacy of indocyanine green fluorescence angiography in preventing anastomotic leakage after laparoscopic colorectal cancer surgery. Int J Colorectal Dis. 2020;35(2):269–75. 31838580 10.1007/s00384-019-03482-0

[83] Tsang YP , Leung LA , Lau CW , Tang CN . Indocyanine green fluorescence angiography to evaluate anastomotic perfusion in colorectal surgery. Int J Colorectal Dis. 2020;35:1133–9. 32291508 10.1007/s00384-020-03592-0

[84] Watanabe J , Ishibe A , Suwa Y , Suwa H , Ota M , Kunisaki C , et al. Indocyanine green fluorescence imaging to reduce the risk of anastomotic leakage in laparoscopic low anterior resection for rectal cancer: a propensity score‐matched cohort study. Surg Endosc. 2020;34(1):202–8. 30877565 10.1007/s00464-019-06751-9

[85] Safiejko K , Tarkowski R , Kozlowski TP , Koselak M , Jachimiuk M , Tarasik A , et al. Safety and efficacy of Indocyanine green in colorectal cancer surgery: a systematic review and meta‐analysis of 11,047 patients. Cancers (Basel). 2022;14(4):1036. 35205784 10.3390/cancers14041036 PMC8869881

[86] Jafari MD , Pigazzi A , McLemore EC , et al. Perfusion assessment in left‐sided/low anterior resection (PILLAR III): a randomized, controlled, parallel, multicenter study assessing perfusion outcomes with PINPOINT near‐infrared fluorescence imaging in low anterior resection. Dis Colon Rectum. 2021;64(8):995–1002. 33872284 10.1097/DCR.0000000000002007

[87] Qu H , Liu Y , Bi DS . Clinical risk factors for anastomotic leakage after laparoscopic anterior resection for rectal cancer: a systematic review and meta‐analysis. Surg Endosc. 2015;29(12):3608–17. 25743996 10.1007/s00464-015-4117-x

[88] Ikeda T , Kumashiro R , Taketani K , Ando K , Kimura Y , Saeki H , et al. Endoscopic evaluation of clinical colorectal anastomotic leakage. J Surg Res. 2015;193(1):126–34. 25103641 10.1016/j.jss.2014.07.009

[89] Senagore A , Lane FR , Lee E , Wexner S , Dujovny N , Sklow B , et al. Bioabsorbable staple line reinforcement in restorative proctectomy and anterior resection: a randomized study. Dis Colon Rectum. 2014;57(3):324–30. 24509454 10.1097/DCR.0000000000000065

[90] Saleh M , Cheruvu MS , Moorthy K , Ahmed AR . Laparoscopic sleeve gastrectomy using a synthetic bioabsorbable staple line reinforcement material: post‐operative complications and 6 year outcomes. Ann Med Surg (Lond). 2016;10:83–7. 27594992 10.1016/j.amsu.2016.08.005 PMC4995473

[91] Hamilton NA , Porembka MR , Johnston FM , Gao F , Strasberg SM , Linehan DC , et al. Mesh reinforcement of pancreatic transection decreases incidence of pancreatic occlusion failure for left pancreatectomy: a single‐blinded, randomized controlled trial. Ann Surg. 2012;255(6):1037–42. 22534422 10.1097/SLA.0b013e31825659ef PMC3363360

[92] Geng HZ , Nasier D , Liu B , Gao H , Xu YK . Meta‐analysis of elective surgical complications related to defunctioning loop ileostomy compared with loop colostomy after low anterior resection for rectal carcinoma. Ann R Coll Surg Engl. 2015;97(7):494–501. 26274752 10.1308/003588415X14181254789240 PMC5210131

[93] Matthiessen P , Hallbook O , Rutegard J , Simert G , Sjodahl R . Defunctioning stoma reduces symptomatic anastomotic leakage after low anterior resection of the rectum for cancer: a randomized multicenter trial. Ann Surg. 2007;246(2):207–14. 17667498 10.1097/SLA.0b013e3180603024 PMC1933561

[94] Shiomi A , Ito M , Maeda K , Kinugasa Y , Ota M , Yamaue H , et al. Effects of a diverting stoma on symptomatic anastomotic leakage after low anterior resection for rectal cancer: a propensity score matching analysis of 1,014 consecutive patients. J Am Coll Surg. 2015;220(2):186–94. 25529899 10.1016/j.jamcollsurg.2014.10.017

[95] Goldberg M , Aukett LK , Carmel J , Fellows J , Pittman J . Management of the patient with a fecal ostomy: best practice guideline for clinicians. J Wound Ostomy Continence Nurs. 2010;37(6):596–8. 21076257 10.1097/WON.0b013e3181f97e37

[96] Miyo M , Takemasa I , Ikeda M , Tujie M , Hasegawa J , Ohue M , et al. The influence of specific technical maneuvers utilized in the creation of diverting loop‐ileostomies on stoma‐related morbidity. Surg Today. 2017;47(8):940–50. 28280983 10.1007/s00595-017-1481-2

[97] Gachabayov M , Lee H , Chudner A , Dyatlov A , Zhang N , Bergamaschi R . Purse‐string vs linear skin closure at loop ileostomy reversal: a systematic review and meta‐analysis. Tech Coloproctol. 2019;23(3):207–20. 30809775 10.1007/s10151-019-01952-9

[98] Mangram AJ , Horan TC , Pearson ML , Silver LC , Jarvis WR . Guideline for prevention of surgical site infection, 1999. Centers for Disease Control and Prevention (CDC) hospital infection control practices advisory committee. Am J Infect Control. 1999;27(2):97–132. 10196487

[99] Okuya K , Takemasa I , Tsuruma T , Noda A , Sasaki K , Ueki T , et al. Evaluation of negative‐pressure wound therapy for surgical site infections after ileostomy closure in colorectal cancer patients: a prospective multicenter study. Surg Today. 2020;50(12):1687–93. 32638132 10.1007/s00595-020-02068-6

[100] Nishigori N , Koyama F , Nakagawa T , et al. Visualization of lymph/blood flow in laparoscopic colorectal cancer surgery by ICG fluorescence imaging (lap‐IGFI). Ann Surg Oncol. 2016;23(Suppl 2):S266–74. 25801355 10.1245/s10434-015-4509-0

[101] Watanabe J , Ota M , Suwa Y , Ishibe A , Masui H , Nagahori K . Real‐time Indocyanine green fluorescence imaging‐guided complete Mesocolic excision in laparoscopic flexural colon cancer surgery. Dis Colon Rectum. 2016;59(7):701–5. 27270525 10.1097/DCR.0000000000000608

[102] Watanabe J , Ota M , Suwa Y , Ishibe A , Masui H , Nagahori K . Evaluation of lymph flow patterns in splenic flexural colon cancers using laparoscopic real‐time indocyanine green fluorescence imaging. Int J Colorectal Dis. 2017;32(2):201–7. 27695977 10.1007/s00384-016-2669-4

[103] Satoyoshi T , Okita K , Ishii M , Hamabe A , Usui A , Akizuki E , et al. Timing of indocyanine green injection prior to laparoscopic colorectal surgery for tumor localization: a prospective case series. Surg Endosc. 2021;35(2):763–9. 32072278 10.1007/s00464-020-07443-5 PMC7819920

[104] Zhou SC , Tian YT , Wang XW , Zhao CD , Ma S , Jiang J , et al. Application of indocyanine green‐enhanced near‐infrared fluorescence‐guided imaging in laparoscopic lateral pelvic lymph node dissection for middle‐low rectal cancer. World J Gastroenterol. 2019;25(31):4502–11. 31496628 10.3748/wjg.v25.i31.4502 PMC6710176

[105] Ohya H , Watanabe J , Suwa H , Suwa Y , Ozawa M , Ishibe A , et al. Near‐infrared imaging using Indocyanine green for laparoscopic lateral pelvic lymph node dissection for clinical stage II/III middle‐lower rectal cancer: a propensity score‐matched cohort study. Dis Colon Rectum. 2022;65(7):885–93. 34840301 10.1097/DCR.0000000000002156

[106] Kelder W , Braat AE , Karrenbeld A , Grond JAK , de Vries JE , Oosterhuis JWA , et al. The sentinel node procedure in colon carcinoma: a multi‐Centre study in The Netherlands. Int J Colorectal Dis. 2007;22(12):1509. 17622543 10.1007/s00384-007-0351-6 PMC2039795

[107] Kakizoe M , Watanabe J , Suwa Y , et al. The histopathological evaluation based on the indocyanine green fluorescence imaging of regional lymph node metastasis of splenic flexural colon cancer by near‐infrared observation. Int J Colorectal Dis. 2020;36:717–23. 33215238 10.1007/s00384-020-03798-2

[108] Sato Y , Satoyoshi T , Okita K , Kyuno D , Hamabe A , Okuya K , et al. Snapshots of lymphatic pathways in colorectal cancer surgery using near‐infrared fluorescence, in vivo and ex vivo. Eur J Surg Oncol. 2021;47(12):3130–6. 34373159 10.1016/j.ejso.2021.07.025

[109] Barnes TG , Penna M , Hompes R , Cunningham C . Fluorescence to highlight the urethra: a human cadaveric study. Tech Coloproctol. 2017;21(6):439–44. 28560481 10.1007/s10151-017-1615-y PMC5495841

[110] Atallah S , Martin‐Perez B , Drake J , Stotland P , Ashamalla S , Albert M . The use of a lighted stent as a method for identifying the urethra in male patients undergoing transanal total mesorectal excision: a video demonstration. Tech Coloproctol. 2015;19(6):375. 25813337 10.1007/s10151-015-1297-2

[111] Swedish Rectal Cancer T , Cedermark B , Dahlberg M , et al. Improved survival with preoperative radiotherapy in resectable rectal cancer. N Engl J Med. 1997;336(14):980–7. 9091798 10.1056/NEJM199704033361402

[112] Kapiteijn EMC , Nagtegaal ID , Putter H , Steup WH , Wiggers T , Rutten HJ , et al. Preoperative radiotherapy combined with total mesorectal excision for resectable rectal cancer. N Engl J Med. 2001;345:638–46. 11547717 10.1056/NEJMoa010580

[113] Bosset JF , Collette L , Calais G , Mineur L , Maingon P , Radosevic‐Jelic L , et al. Chemotherapy with preoperative radiotherapy in rectal cancer. N Engl J Med. 2006;355(11):1114–23. 16971718 10.1056/NEJMoa060829

[114] Gerard JP , Conroy T , Bonnetain F , et al. Preoperative radiotherapy with or without concurrent fluorouracil and leucovorin in T3‐4 rectal cancers: results of FFCD 9203. J Clin Oncol. 2006;24(28):4620–5. 17008704 10.1200/JCO.2006.06.7629

[115] Bonnetain F , Bosset JF , Gerard JP , Calais G , Conroy T , Mineur L , et al. What is the clinical benefit of preoperative chemoradiotherapy with 5FU/leucovorin for T3‐4 rectal cancer in a pooled analysis of EORTC 22921 and FFCD 9203 trials: surrogacy in question? Eur J Cancer. 2012;48(12):1781–90. 22507892 10.1016/j.ejca.2012.03.016

[116] Sauer R , Liersch T , Merkel S , Fietkau R , Hohenberger W , Hess C , et al. Preoperative versus postoperative chemoradiotherapy for locally advanced rectal cancer: results of the German CAO/ARO/AIO‐94 randomized phase III trial after a median follow‐up of 11 years. J Clin Oncol. 2012;30(16):1926–33. 22529255 10.1200/JCO.2011.40.1836

[117] Hashiguchi Y , Muro K , Saito Y , Ito Y , Ajioka Y , Hamaguchi T , et al. Japanese Society for Cancer of the colon and Rectum (JSCCR) guidelines 2019 for the treatment of colorectal cancer. Int J Clin Oncol. 2020;25(1):1–42. 31203527 10.1007/s10147-019-01485-z PMC6946738

[118] Stevenson ARL , Solomon MJ , Brown CSB , Lumley JW , Hewett P , Clouston AD , et al. Disease‐free survival and local recurrence after laparoscopic‐assisted resection or open resection for rectal cancer: the Australasian laparoscopic cancer of the rectum randomized clinical trial. Ann Surg. 2019;269(4):596–602. 30247332 10.1097/SLA.0000000000003021

[119] Fleshman J , Branda ME , Sargent DJ , Boller AM , George VV , Abbas MA , et al. Disease‐free survival and local recurrence for laparoscopic resection compared with open resection of stage II to III rectal cancer: follow‐up results of the ACOSOG Z6051 randomized controlled trial. Ann Surg. 2019;269(4):589–95. 30080730 10.1097/SLA.0000000000003002 PMC6360134

[120] Petrelli F , Trevisan F , Cabiddu M , Sgroi G , Bruschieri L , Rausa E , et al. Total neoadjuvant therapy in rectal cancer: a systematic review and meta‐analysis of treatment outcomes. Ann Surg. 2020;271(3):440–8. 31318794 10.1097/SLA.0000000000003471

[121] Bujko K , Wyrwicz L , Rutkowski A , Malinowska M , Pietrzak L , Kryński J , et al. Long‐course oxaliplatin‐based preoperative chemoradiation versus 5 x 5 Gy and consolidation chemotherapy for cT4 or fixed cT3 rectal cancer: results of a randomized phase III study. Ann Oncol. 2016;27(5):834–42. 26884592 10.1093/annonc/mdw062

[122] Cisel B , Pietrzak L , Michalski W , et al. Long‐course preoperative chemoradiation versus 5 x 5 Gy and consolidation chemotherapy for clinical T4 and fixed clinical T3 rectal cancer: long‐term results of the randomized polish II study. Ann Oncol. 2019;30(8):1298–303. 31192355 10.1093/annonc/mdz186

[123] Jin J , Tang Y , Hu C , et al. Multicenter, randomized, phase III trial of short‐term radiotherapy plus chemotherapy versus long‐term Chemoradiotherapy in locally advanced rectal cancer (STELLAR). J Clin Oncol. 2022;40:1681–92. 35263150 10.1200/JCO.21.01667 PMC9113208

[124] Bahadoer RR , Dijkstra EA , van Etten B , Marijnen CAM , Putter H , Kranenbarg EMK , et al. Short‐course radiotherapy followed by chemotherapy before total mesorectal excision (TME) versus preoperative chemoradiotherapy, TME, and optional adjuvant chemotherapy in locally advanced rectal cancer (RAPIDO): a randomised, open‐label, phase 3 trial. Lancet Oncol. 2021;22(1):29–42. 33301740 10.1016/S1470-2045(20)30555-6

[125] Conroy T , Bosset J‐F , Etienne P‐L , Rio E , François É , Mesgouez‐Nebout N , et al. Neoadjuvant chemotherapy with FOLFIRINOX and preoperative chemoradiotherapy for patients with locally advanced rectal cancer (UNICANCER‐PRODIGE 23): a multicentre, randomised, open‐label, phase 3 trial. Lancet Oncol. 2021;22(5):702–15. 33862000 10.1016/S1470-2045(21)00079-6

[126] Smith JJ , Strombom P , Chow OS , Roxburgh CS , Lynn P , Eaton A , et al. Assessment of a watch‐and‐wait strategy for rectal cancer in patients with a complete response after neoadjuvant therapy. JAMA Oncol. 2019;5(4):e185896. 30629084 10.1001/jamaoncol.2018.5896 PMC6459120

[127] van der Valk MJM , Hilling DE , Bastiaannet E , Meershoek‐Klein Kranenbarg E , Beets GL , Figueiredo NL , et al. Long‐term outcomes of clinical complete responders after neoadjuvant treatment for rectal cancer in the International Watch & Wait Database (IWWD): an international multicentre registry study. Lancet. 2018;391(10139):2537–45. 29976470 10.1016/S0140-6736(18)31078-X

[128] Beard BW , Rettig RL , Ryoo JJ , Parker RA , McLemore EC , Attaluri V . Watch‐and‐wait compared to operation for patients with complete response to neoadjuvant therapy for rectal cancer. J Am Coll Surg. 2020;231(6):681–92. 33121903 10.1016/j.jamcollsurg.2020.08.775

[129] Garcia‐Aguilar J , Patil S , Gollub MJ , et al. Organ preservation in patients with rectal adenocarcinoma treated with Total neoadjuvant therapy. J Clin Oncol. 2022;40:2546–56. 35483010 10.1200/JCO.22.00032 PMC9362876

[130] Birgisson H , Pahlman L , Gunnarsson U , Glimelius B . Occurrence of second cancers in patients treated with radiotherapy for rectal cancer. J Clin Oncol. 2005;23(25):6126–31. 16135478 10.1200/JCO.2005.02.543

[131] Birgisson H , Pahlman L , Gunnarsson U , Glimelius B , Swedish Rectal Cancer Trial G . Adverse effects of preoperative radiation therapy for rectal cancer: long‐term follow‐up of the Swedish rectal cancer trial. J Clin Oncol. 2005;23(34):8697–705. 16314629 10.1200/JCO.2005.02.9017

[132] Marijnen CA , Kapiteijn E , van de Velde CJ , et al. Acute side effects and complications after short‐term preoperative radiotherapy combined with total mesorectal excision in primary rectal cancer: report of a multicenter randomized trial. J Clin Oncol. 2002;20(3):817–25. 11821466 10.1200/JCO.2002.20.3.817

[133] Peeters KC , van de Velde CJ , Leer JW , et al. Late side effects of short‐course preoperative radiotherapy combined with total mesorectal excision for rectal cancer: increased bowel dysfunction in irradiated patients–a Dutch colorectal cancer group study. J Clin Oncol. 2005;23(25):6199–206. 16135487 10.1200/JCO.2005.14.779

[134] Deng Y , Chi P , Lan P , Wang L , Chen W , Cui L , et al. Modified FOLFOX6 with or without radiation versus fluorouracil and Leucovorin with radiation in neoadjuvant treatment of locally advanced rectal cancer: initial results of the Chinese FOWARC multicenter, open‐label, randomized three‐arm phase III trial. J Clin Oncol. 2016;34(27):3300–7. 27480145 10.1200/JCO.2016.66.6198

[135] Deng Y , Chi P , Lan P , Wang L , Chen W , Cui L , et al. Neoadjuvant modified FOLFOX6 with or without radiation versus fluorouracil plus radiation for locally advanced rectal cancer: final results of the Chinese FOWARC trial. J Clin Oncol. 2019;37(34):3223–33. 31557064 10.1200/JCO.18.02309 PMC6881102

[136] Hasegawa J , Nishimura J , Mizushima T , Miyake Y , Kim HM , Takemoto H , et al. Neoadjuvant capecitabine and oxaliplatin (XELOX) combined with bevacizumab for high‐risk localized rectal cancer. Cancer Chemother Pharmacol. 2014;73(5):1079–87. 24595805 10.1007/s00280-014-2417-9

[137] Hata T , Takahashi H , Sakai D , Haraguchi N , Nishimura J , Kudo T , et al. Neoadjuvant CapeOx therapy followed by sphincter‐preserving surgery for lower rectal cancer. Surg Today. 2017;47(11):1372–7. 28474202 10.1007/s00595-017-1527-5

[138] Kudo T , Takemasa I , Hata T , Sakai D , Takahashi H , Haraguchi N , et al. A phase I study of neoadjuvant Capecitabine, Oxaliplatin, and irinotecan (XELOXIRI) in patients with locally advanced rectal cancer. Oncology. 2019;97(4):211–6. 31266024 10.1159/000500677

[139] Kamiya T , Uehara K , Nakayama G , Ishigure K , Kobayashi S , Hiramatsu K , et al. Early results of multicenter phase II trial of perioperative oxaliplatin and capecitabine without radiotherapy for high‐risk rectal cancer: CORONA I study. Eur J Surg Oncol. 2016;42(6):829–35. 26968228 10.1016/j.ejso.2016.02.014

[140] Uehara K , Hiramatsu K , Maeda A , Sakamoto E , Inoue M , Kobayashi S , et al. Neoadjuvant oxaliplatin and capecitabine and bevacizumab without radiotherapy for poor‐risk rectal cancer: N‐SOG 03 phase II trial. Jpn J Clin Oncol. 2013;43(10):964–71. 23935207 10.1093/jjco/hyt115

[141] Cercek A , Lumish M , Sinopoli J , Weiss J , Shia J , Lamendola‐Essel M , et al. PD‐1 blockade in mismatch repair‐deficient, locally advanced rectal cancer. N Engl J Med. 2022;386(25):2363–76. 35660797 10.1056/NEJMoa2201445 PMC9492301

[142] Abe T , Yasui M , Imamura H , Matsuda C , Nishimura J , Haraguchi N , et al. Combination of extramural venous invasion and lateral lymph node size detected with magnetic resonance imaging is a reliable biomarker for lateral lymph node metastasis in patients with rectal cancer. World J Surg Oncol. 2022;20(1):5. 34986842 10.1186/s12957-021-02464-3 PMC8728915

[143] Agger E , Akerlund V , Ekberg O , Jorgren F , Lydrup ML , Buchwald P . Management, treatment and prognostic significance of lateral lymph node metastases in rectal cancer‐a regional cohort study. Int J Colorectal Dis. 2021;36(12):2707–14. 34487231 10.1007/s00384-021-04018-1 PMC8589806

[144] Noura S , Yamamoto H , Ohnishi T , Masuda N , Matsumoto T , Takayama O , et al. Comparative detection of lymph node micrometastases of stage II colorectal cancer by reverse transcriptase polymerase chain reaction and immunohistochemistry. J Clin Oncol. 2002;20(20):4232–41. 12377967 10.1200/JCO.2002.10.023

[145] Tani K , Itabashi M , Okuya K , Okita K , Takemasa I , Tomita N , et al. Feasibility of pooled one‐step nucleic acid amplification for molecular staging of pathologically node‐negative colon cancer: a prospective multicenter study. Ann Surg Oncol. 2021;28(13):8804–12. 34086123 10.1245/s10434-021-10140-9

[146] Miyake Y , Mizushima T , Hata T , Takahashi H , Hanada H , Shoji H , et al. Inspection of perirectal lymph nodes by one‐step nucleic acid amplification predicts lateral lymph node metastasis in advanced rectal cancer. Ann Surg Oncol. 2017;24(13):3850–6. 28924845 10.1245/s10434-017-6069-y

[147] Siravegna G , Mussolin B , Buscarino M , Corti G , Cassingena A , Crisafulli G , et al. Clonal evolution and resistance to EGFR blockade in the blood of colorectal cancer patients. Nat Med. 2015;21(7):795–801. 26030179 10.1038/nm.3870 PMC4868598

[148] Diehl F , Schmidt K , Choti MA , Romans K , Goodman S , Li M , et al. Circulating mutant DNA to assess tumor dynamics. Nat Med. 2008;14(9):985–90. 18670422 10.1038/nm.1789 PMC2820391

[149] Abbosh C , Birkbak NJ , Wilson GA , et al. Phylogenetic ctDNA analysis depicts early‐stage lung cancer evolution. Nature. 2017;545(7655):446–51. 28445469 10.1038/nature22364 PMC5812436

[150] Nakamura Y , Taniguchi H , Ikeda M , Bando H , Kato K , Morizane C , et al. Clinical utility of circulating tumor DNA sequencing in advanced gastrointestinal cancer: SCRUM‐Japan GI‐SCREEN and GOZILA studies. Nat Med. 2020;26(12):1859–64. 33020649 10.1038/s41591-020-1063-5

[151] Tie J , Wang Y , Tomasetti C , et al. Circulating tumor DNA analysis detects minimal residual disease and predicts recurrence in patients with stage II colon cancer. Sci Transl Med. 2016;8(346):346ra392. 10.1126/scitranslmed.aaf6219 PMC5346159 27384348

[152] Reinert T , Henriksen TV , Christensen E , Sharma S , Salari R , Sethi H , et al. Analysis of plasma cell‐free DNA by Ultradeep sequencing in patients with stages I to III colorectal cancer. JAMA Oncol. 2019;5(8):1124–31. 31070691 10.1001/jamaoncol.2019.0528 PMC6512280

[153] Taniguchi H , Nakamura Y , Kotani D , Yukami H , Mishima S , Sawada K , et al. CIRCULATE‐Japan: circulating tumor DNA‐guided adaptive platform trials to refine adjuvant therapy for colorectal cancer. Cancer Sci. 2021;112(7):2915–20. 33931919 10.1111/cas.14926 PMC8253296

[154] Grothey A , Sobrero AF , Shields AF , Yoshino T , Paul J , Taieb J , et al. Duration of adjuvant chemotherapy for stage III colon cancer. N Engl J Med. 2018;378(13):1177–88. 29590544 10.1056/NEJMoa1713709 PMC6426127

[155] Yoshino T , Yamanaka T , Oki E , Kotaka M , Manaka D , Eto T , et al. Efficacy and long‐term peripheral sensory neuropathy of 3 vs 6 mos of Oxaliplatin‐based adjuvant chemotherapy for colon cancer: the ACHIEVE phase 3 randomized clinical trial. JAMA Oncol. 2019;5(11):1574–81. 31513248 10.1001/jamaoncol.2019.2572 PMC6743062

[156] Tie J , Cohen JD , Lahouel K , Lo SN , Wang Y , Kosmider S , et al. Circulating tumor DNA analysis guiding adjuvant therapy in stage II colon cancer. N Engl J Med. 2022;386(24):2261–72. 35657320 10.1056/NEJMoa2200075 PMC9701133

[157] Pimentel‐Nunes P , Dinis‐Ribeiro M , Ponchon T , Repici A , Vieth M , de Ceglie A , et al. Endoscopic submucosal dissection: European Society of Gastrointestinal Endoscopy (ESGE) guideline. Endoscopy. 2015;47(9):829–54. 26317585 10.1055/s-0034-1392882

[158] Miyo M , Kato T , Nakamura Y , Taniguchi H , Takahashi Y , Ishii M , et al. DENEB: development of new criteria for curability after local excision of pathological T1 colorectal cancer using liquid biopsy. Cancer Sci. 2022;113(4):1531–4. 34839585 10.1111/cas.15226 PMC8990725

[159] Tie J , Cohen JD , Wang Y , Li L , Christie M , Simons K , et al. Serial circulating tumour DNA analysis during multimodality treatment of locally advanced rectal cancer: a prospective biomarker study. Gut. 2019;68(4):663–71. 29420226 10.1136/gutjnl-2017-315852 PMC6265124

[160] Zhou J , Wang C , Lin G , Xiao Y , Jia W , Xiao G , et al. Serial circulating tumor DNA in predicting and monitoring the effect of neoadjuvant Chemoradiotherapy in patients with rectal cancer: a prospective multicenter study. Clin Cancer Res. 2021;27(1):301–10. 33046514 10.1158/1078-0432.CCR-20-2299

[161] Takemasa I , Hamabe A , Ishii M . Perspectives for circulating tumor DNA in clinical management of colorectal cancer. Int J Clin Oncol. 2021;26(8):1420–30. 34185174 10.1007/s10147-021-01937-5

[162] Brown G , Daniels IR , Richardson C , Revell P , Peppercorn D , Bourne M . Techniques and trouble‐shooting in high spatial resolution thin slice MRI for rectal cancer. Br J Radiol. 2005;78(927):245–51. 15730990 10.1259/bjr/33540239

[163] Nougaret S , Rousset P , Gormly K , et al. Structured and shared MRI staging lexicon and report of rectal cancer: a consensus proposal by the French radiology Group (GRERCAR) and surgical Group (GRECCAR) for rectal cancer. Diagn Interv Imaging. 2021;103:127–41. 34794932 10.1016/j.diii.2021.08.003

[164] Taylor FG , Quirke P , Heald RJ , Moran BJ , Blomqvist L , Swift IR , et al. Preoperative magnetic resonance imaging assessment of circumferential resection margin predicts disease‐free survival and local recurrence: 5‐year follow‐up results of the MERCURY study. J Clin Oncol. 2014;32(1):34–43. 24276776 10.1200/JCO.2012.45.3258

[165] Battersby NJ , How P , Moran B , Stelzner S , West NP , Branagan G , et al. Prospective validation of a low rectal cancer magnetic resonance imaging staging system and development of a local recurrence risk stratification model: the MERCURY II study. Ann Surg. 2016;263(4):751–60. 25822672 10.1097/SLA.0000000000001193

[166] Group MS . Extramural depth of tumor invasion at thin‐section MR in patients with rectal cancer: results of the MERCURY study. Radiology. 2007;243(1):132–9. 17329685 10.1148/radiol.2431051825

[167] Trebeschi S , van Griethuysen JJM , Lambregts DMJ , Lahaye MJ , Parmar C , Bakers FCH , et al. Deep learning for fully‐automated localization and segmentation of rectal cancer on multiparametric MR. Sci Rep. 2017;7(1):5301. 28706185 10.1038/s41598-017-05728-9 PMC5509680

[168] Huang YJ , Dou Q , Wang ZX , Liu LZ , Jin Y , Li CF , et al. 3‐D RoI‐aware U‐net for accurate and efficient colorectal tumor segmentation. IEEE Trans Cybern. 2021;51(11):5397–408. 32248143 10.1109/TCYB.2020.2980145

[169] Hamabe A , Ishii M , Kamoda R , Sasuga S , Okuya K , Okita K , et al. Artificial intelligence–based technology for semi‐automated segmentation of rectal cancer using high‐resolution MRI. Plos One. 2022;17(6):e0269931. 35714069 10.1371/journal.pone.0269931 PMC9205476

[170] Hamabe A , Ishii M , Kamoda R , Sasuga S , Okuya K , Okita K , et al. Artificial intelligence‐based technology to make a three‐dimensional pelvic model for preoperative simulation of rectal cancer surgery using MRI. Ann Gastroenterol Surg. 2022;6:788–94. 36338585 10.1002/ags3.12574 PMC9628238

[171] Bryant CL , Lunniss PJ , Knowles CH , Thaha MA , Chan CL . Anterior resection syndrome. Lancet Oncol. 2012;13(9):e403–8. 22935240 10.1016/S1470-2045(12)70236-X

[172] Keane C , Wells C , O'Grady G , Bissett IP . Defining low anterior resection syndrome: a systematic review of the literature. Colorectal Dis. 2017;19(8):713–22. 28612460 10.1111/codi.13767

[173] Chapman SJ , Bolton WS , Corrigan N , Young N , Jayne DG . A cross‐sectional review of reporting variation in postoperative bowel dysfunction after rectal cancer surgery. Dis Colon Rectum. 2017;60(2):240–7. 28059921 10.1097/DCR.0000000000000649

[174] Emmertsen KJ , Laurberg S . Low anterior resection syndrome score: development and validation of a symptom‐based scoring system for bowel dysfunction after low anterior resection for rectal cancer. Ann Surg. 2012;255(5):922–8. 22504191 10.1097/SLA.0b013e31824f1c21

[175] Juul T , Ahlberg M , Biondo S , Emmertsen KJ , Espin E , Jimenez LM , et al. International validation of the low anterior resection syndrome score. Ann Surg. 2014;259(4):728–34. 23598379 10.1097/SLA.0b013e31828fac0b

[176] Juul T , Ahlberg M , Biondo S , Espin E , Jimenez LM , Matzel KE , et al. Low anterior resection syndrome and quality of life: an international multicenter study. Dis Colon Rectum. 2014;57(5):585–91. 24819098 10.1097/DCR.0000000000000116

[177] Kupsch J , Kuhn M , Matzel KE , Zimmer J , Radulova‐Mauersberger O , Sims A , et al. To what extent is the low anterior resection syndrome (LARS) associated with quality of life as measured using the EORTC C30 and CR38 quality of life questionnaires? Int J Colorectal Dis. 2019;34(4):747–62. 30721417 10.1007/s00384-019-03249-7

[178] Akizuki E , Okita K , Noda A , Tsuruma T , Nishimori H , Sasaki K , et al. Clinical utility and characteristics of the LARS score compared to the CCIS. World J Surg. 2022;46(4):925–32. 35119510 10.1007/s00268-021-06405-9

[179] Akizuki E , Matsuno H , Satoyoshi T , Ishii M , Usui A , Ueki T , et al. Validation of the Japanese version of the low anterior resection syndrome score. World J Surg. 2018;42(8):2660–7. 29450698 10.1007/s00268-018-4519-8 PMC6060820

[180] Keane C , Sharma P , Yuan L , Bissett I , O'Grady G . Impact of temporary ileostomy on long‐term quality of life and bowel function: a systematic review and meta‐analysis. ANZ J Surg. 2020;90(5):687–92. 31701636 10.1111/ans.15552

[181] Battersby NJ , Bouliotis G , Emmertsen KJ , Juul T , Glynne‐Jones R , Branagan G , et al. Development and external validation of a nomogram and online tool to predict bowel dysfunction following restorative rectal cancer resection: the POLARS score. Gut. 2018;67(4):688–96. 28115491 10.1136/gutjnl-2016-312695

[182] Korai T , Akizuki E , Okita K , Nishidate T , Okuya K , Sato Y , et al. Defecation disorder and anal function after surgery for lower rectal cancer in elderly patients. Ann Gastroenterol Surg. 2022;6(1):101–8. 35106420 10.1002/ags3.12505 PMC8786691

[183] Shirouzu K , Murakami N , Akagi Y . Intersphincteric resection for very low rectal cancer: a review of the updated literature. Ann Gastroenterol Surg. 2017;1(1):24–32. 29863144 10.1002/ags3.12003 PMC5881339

[184] Christensen P , Im Baeten C , Espín‐Basany E , et al. Management guidelines for low anterior resection syndrome – the MANUEL project. Colorectal Dis. 2021;23(2):461–75. 33411977 10.1111/codi.15517 PMC7986060

[185] Keane C , Fearnhead NS , Bordeianou LG , Christensen P , Basany EE , Laurberg S , et al. International consensus definition of low anterior resection syndrome. Dis Colon Rectum. 2020;63(3):274–84. 32032141 10.1097/DCR.0000000000001583 PMC7034376

[186] Lange MM , van de Velde CJ . Urinary and sexual dysfunction after rectal cancer treatment. Nat Rev Urol. 2011;8(1):51–7. 21135876 10.1038/nrurol.2010.206

[187] Ito M , Kobayashi A , Fujita S , Mizusawa J , Kanemitsu Y , Kinugasa Y , et al. Urinary dysfunction after rectal cancer surgery: results from a randomized trial comparing mesorectal excision with and without lateral lymph node dissection for clinical stage II or III lower rectal cancer (Japan clinical oncology Group study, JCOG0212). Eur J Surg Oncol. 2018;44(4):463–8. 29428473 10.1016/j.ejso.2018.01.015

[188] Hamamoto H , Yamamoto M , Masubuchi S , Ishii M , Osumi W , Tanaka K , et al. Male sex and anterior wall tumor location as risk factors for urinary dysfunction after laparoscopic rectal surgery. Surg Endosc. 2020;34(8):3567–73. 31605220 10.1007/s00464-019-07186-y

[189] Kinugasa Y , Murakami G , Suzuki D , Sugihara K . Histological identification of fascial structures posterolateral to the rectum. Br J Surg. 2007;94(5):620–6. 17330242 10.1002/bjs.5540

[190] Emile SH , Elfeki H , Shalaby M , Sakr A , Kim NK . Outcome of lateral pelvic lymph node dissection with total mesorectal excision in treatment of rectal cancer: a systematic review and meta‐analysis. Surgery. 2021;169(5):1005–15. 33317903 10.1016/j.surg.2020.11.010

[191] Georgiou P , Tan E , Gouvas N , Antoniou A , Brown G , Nicholls RJ , et al. Extended lymphadenectomy versus conventional surgery for rectal cancer: a meta‐analysis. Lancet Oncol. 2009;10(11):1053–62. 19767239 10.1016/S1470-2045(09)70224-4

[192] Lee SY , Kang SB , Kim DW , Oh HK , Ihn MH . Risk factors and preventive measures for acute urinary retention after rectal cancer surgery. World J Surg. 2015;39(1):275–82. 25189452 10.1007/s00268-014-2767-9

[193] Kim HO , Cho YS , Kim H , Lee SR , Jung KU , Chun HK . Scoring systems used to predict bladder dysfunction after laparoscopic rectal cancer surgery. World J Surg. 2016;40(12):3044–51. 27456498 10.1007/s00268-016-3636-5

[194] Lange MM , Marijnen CA , Maas CP , et al. Risk factors for sexual dysfunction after rectal cancer treatment. Eur J Cancer. 2009;45(9):1578–88. 19147343 10.1016/j.ejca.2008.12.014

[195] Yamaoka Y , Kagawa H , Shiomi A , Yamakawa Y , Hino H , Manabe S , et al. Robotic‐assisted surgery may be a useful approach to protect urinary function in the modern era of diverse surgical approaches for rectal cancer. Surg Endosc. 2021;35(3):1317–23. 32215747 10.1007/s00464-020-07509-4

[196] Miura T , Sakamoto Y , Morohashi H , Suto A , Kubota S , Ichisawa A , et al. Robotic surgery contributes to the preservation of bowel and urinary function after total mesorectal excision: comparisons with transanal and conventional laparoscopic surgery. BMC Surg. 2022;22(1):147. 35449005 10.1186/s12893-022-01596-x PMC9026934

[197] Galata C , Vassilev G , Haas F , Kienle P , Büttner S , Reißfelder C , et al. Clinical, oncological, and functional outcomes of Da Vinci (xi)‐assisted versus conventional laparoscopic resection for rectal cancer: a prospective, controlled cohort study of 51 consecutive cases. Int J Colorectal Dis. 2019;34(11):1907–14. 31642968 10.1007/s00384-019-03397-w

